# Fumarate inhibits the formation of neutrophil extracellular traps (NETs) in a Nrf2-controlled and Annexin-A1-dependent manner associated with mitochondrial fusion

**DOI:** 10.3389/fimmu.2026.1770063

**Published:** 2026-03-31

**Authors:** Gabriela Burczyk, Elzbieta Kolaczkowska

**Affiliations:** 1 Department of Experimental Hematology, Institute of Zoology and Biomedical Research, Faculty of Biology, Jagiellonian University, Krakow, Poland; 2 Doctoral School of Exact and Natural Sciences, Faculty of Biology, Jagiellonian University, Krakow, Poland

**Keywords:** Annexin A1, ANXA1, fumarate, mitochondria fission, mitochondria fusion, neutrophils, Nrf2, PANoptosis

## Abstract

Neutrophil extracellular traps (NETs) constitute a critical antimicrobial mechanism, yet excessive or dysregulated NET release contributes to endothelial injury and tissue damage. Therefore, identifying physiological and pharmacological regulators of NET formation remains an important goal. Although the role of mitochondrial dynamics in NETs remains incompletely elucidated, accumulating evidence suggests that mitochondria may be underexplored regulators with therapeutic potential. In fact, in certain NET forms, their DNA is of mitochondrial origin. Here, we investigated how exogenous dimethyl fumarate (DMF), an ester of the tricarboxylic acid cycle (TCA) metabolite fumarate, modulates NET formation. Foremost, we observed that DMF markedly suppresses PAD4-dependent NET release by LPS-stimulated neutrophils of wild-type and PAD4-deficient mice. Mechanistic analyses demonstrated that DMF activates the nuclear factor erythroid 2-related factor 2 (Nrf2) pathway and increases the secretion of anti-inflammatory Annexin A1 (ANXA1). Functionally, inhibition of either Nrf2 or the ANXA1 receptor Fpr2 restores NET formation. To integrate these observations with mitochondrial function, we examined markers of mitochondrial dynamics. We found that DMF decreases phosphorylation of dynamin-related protein 1 (DRP1) at Serine 616, a modification typically associated with reduced mitochondrial fission. Consistently, pharmacological inhibition of DRP1 (Mdivi-1) also diminishes NET formation, whereas induction of mitochondrial fragmentation (CCCP) triggers PANoptotic neutrophil death and extracellular DNA release, both of which were prevented by DMF. Collectively, these data identify DMF as a mitochondria-linked immunometabolic regulator that suppresses NET formation through coordinated engagement of Nrf2 and ANXA1 signaling and modulation of mitochondrial dynamics. These findings highlight mitochondrial remodeling as a promising avenue for future exploration and position DMF as a potential pharmacological tool for controlling excessive neutrophil activation.

## Graphical Abstract

**Figure:**
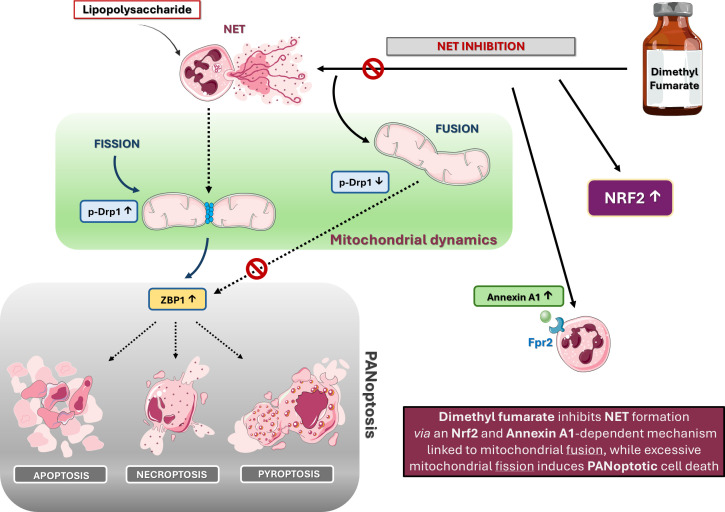

## Introduction

Neutrophils release neutrophil extracellular traps (NETs) upon strong activation by exogenous stimuli such as lipopolysaccharides (LPS) or whole bacteria (1), but also in response to endogenous inflammatory factors, including cytokines (2), mitochondrial DNA (3), activated platelets (4) and synthetic agents like phorbol 12-myristate 13-acetate (PMA) (5) or ionomycin (6). NETs consist of an extracellular DNA scaffold decorated with granule-derived proteins (notably neutrophil elastase (NE) and myeloperoxidase) and nuclear histones (7). Early in inflammation, NETs immobilize pathogens and restrict their spread throughout the body (2), however, when they are not cleared efficiently, as seen in sepsis (8), COVID-19 (9), or other severe inflammatory conditions (10, 11) they drive bystander tissue injury, and contribute to multiple organ dysfunction syndromes (MODS) (e.g., liver and kidney failure) (8, 12). Our data further indicate that impaired NET clearance can persist for months, and even when they are eventually removed, new waves of NET formation recur in a self-renewal manner (13).

For these reasons, strategies that limit NET formation (pharmacological or metabolic) are of critical importance. Our research focuses explicitly on how immunometabolites (endogenous metabolic by-products that increase in immune cells in response to their activation (14)), either endogenous molecules or their derivatives, could be used to achieve this. By now, numerous immunometabolites have been characterized as anti-inflammatory; however, most of the studies have centered on macrophages (15) and lymphocytes (16), with comparatively less attention to neutrophils. Nevertheless, emerging evidence indicates that specific metabolites can directly modulate neutrophil activation, underscoring their potential as modulators of neutrophil-driven pathology. For example, treatment with the metabolite of tryptophan catabolism - kynurenic acid (KYNA), reduces NET formation in septic rats (17), and so does sodium butyrate (a metabolite of gut microbiota) in human cells (18). On the other hand, some metabolites do not exert exclusively anti-inflammatory effects, e.g., succinate, the metabolite of the tricarboxylic acid cycle (TCA) – signaling, has been shown to enhance NETs ejection in experimental autoimmune uveitis (19). This underscores the importance of selecting the appropriate immunometabolite for therapeutic modulation of NETs.

Thus far, we have demonstrated (20) that one of the most promising immunometabolites, itaconic acid, produced during “broken Krebs cycle” - characterized initially as an anti-inflammatory mediator in macrophages (21), it also exerts broader immunomodulatory effects in neutrophils. Its mode of action is of importance. O’Neill’s group showed that the itaconate derivative 4-octyl itaconate (4-OI) activates the transcription of the anti-inflammatory nuclear factor erythroid 2-related factor 2 (Nrf2) in macrophages by alkylating the cytosolic sensor - kelch-like ECH-associated protein 1 (KEAP1) (15). Importantly, we have shown that 4-OI suppresses NET formation, and this effect also depends on Nrf2 activation, in addition to increased/heme oxygenase-1 (HO-1) but decreased hypoxia-inducible factor-1α (HIF-1α) (20). This strongly indicates that manipulation of Nrf2 activity might be an essential goal in NET inhibition studies.

For this, in the current study, we selected another TCA cycle-derived metabolite – fumarate (22). Fumaric acid (DMF; a cell-permeable ester of fumarate) has been reported to exert anti-inflammatory effects in macrophages *via* activation of Nrf2 and promotion of secretion of the anti-inflammatory mediator Annexin A1, in a process requiring the cholesterol transporter ABCA1 (23).

Additionally, because endogenous fumarate is generated in the mitochondria during the TCA cycle (22), and because accumulating evidence indicates that mitochondrial status shapes inflammatory responses (24), we considered whether DMF might modulate mitochondrial activity in neutrophils, which could impact NET formation. Mitochondria are highly dynamic organelles undergoing constant cycles of fusion and fission (fragmentation), and the balance between these opposing processes is a key determinant of cellular function (25). Mitochondrial fusion, orchestrated primarily by mitofusins (MFN1, MFN2) and optic atrophy protein 1 (OPA1) (26), facilitates the exchange of mitochondrial contents, dilution of damage, and maintenance of mitochondrial integrity (27). Through these actions, fusion supports efficient oxidative phosphorylation (28) and helps sustain cellular function under stress (24), all of which are generally also associated with the anti-inflammatory state. Conversely, mitochondrial fission (fragmentation), driven mainly by dynamin-related protein 1 (DRP1) and its adaptor proteins, contributes to mitochondrial quality control by segregating dysfunctional segments for removal (25). Yet when excessive, fission increases ROS production, promotes metabolic disruption, and can trigger apoptosis under severe stress (29, 30). Notably, pathological fragmentation is associated with the release of mitochondrial DNA (mtDNA) (31), a potent pro-inflammatory signal implicated in certain forms of NET formation (32). Hence, in studies of DMF, we also asked whether shifts in mitochondrial dynamics alter neutrophil susceptibility to NET formation and whether DMF might shape this process by affecting mitochondrial fusion or fission.

The above questions are particularly relevant because mitochondria regulate both oxidative stress and multiple forms of regulated cell death (RCD) that intersect with NET biology (33). Importantly, NET release is not a uniform process: NET formation might lead to cell death (the process previously known as lytic NETosis), which involves membrane rupture and is closely linked to ROS-dependent signaling (34). On the other hand, numerous neutrophils survive NET release (previously: vital NETosis), remain viable and functionally active; such cells transport NET components to the cell surface *via* dedicated vesicles (35). Understanding how mitochondrial signals contribute to these distinct NET pathways is therefore essential for determining whether mitochondrial-targeting immunometabolites, such as DMF, could be leveraged to modulate NET formation and inflammatory outcomes.

Herein, we report that the fumarate derivative dimethyl fumarate (DMF) strongly inhibits NET formation in an Nrf2-dependent manner that also involves Annexin A1, and that the DMF mode of action is related to mitochondrial fusion. During the experiments, we also observed that strong mitochondrial fission (induced by carbonyl cyanide m-chlorophenylhydrazone, CCCP) leads to cell death involving PANosis. This is a newly identified, highly inflammatory type of programmed cell death that combines features of apoptosis, necroptosis, and pyroptosis, and plays a crucial role in infectious diseases, cancer, and inflammatory conditions (36). Hence, fusion and fragmentation of mitochondria literally decide about a neutrophil’s life or death, respectively. Additionally, mitochondrial fusion promotes the anti-inflammatory state of those cells. Critically, Nrf2 transcription factor turns out to be one of the main drivers controlling NET formation by neutrophils.

## Materials and methods

### Mice

C57BL/6J male mice purchased from Charles River Laboratories (Sulzfeld, Germany; *via* AnimaLab). Peptidyl arginine deiminase 4 (*Pad4*^-/-^) knockout (KO) mice on the C57BL/6J background were purchased from the Jackson Laboratory (Bar Harbor, ME, USA) and they were bred in the Laboratory of Experimental Hematology, Jagiellonian University; C57BL/6J served as their wild-type controls (WT). Animals were housed at a constant temperature (21-22°C) and under the conditions of a daily cycle of 12 h light/12 h darkness. The pelleted food (Altromin, Lage, Germany, C1324, Fat 11%, Carbohydrates 65%, Protein 24% of kcal) and tap water were available *ad libitum*. The Local Ethical Committee approved all procedures for experimental animals No. II in Krakow (22/2023, 22A/2023 and 245/2023) and complied with the EU Animal Care Guidelines.

### Bone marrow derived neutrophil isolation

Neutrophils were isolated from the bone marrow of mice as described previously (20). Briefly, following anesthesia with a mixture of ketamine hydrochloride (Biowet Pulawy, Pulawy, Poland) solution at a dose of 200 mg/kg body weight and xylazine hydrochloride (antiMEDICA, Südfeld, Germany) at a dose of 10 mg/kg body weight and subsequent cervical dislocation, tibias and femurs were collected, cleaned, and flushed with ice-cold Hanks’ Balanced Salt Solution without calcium and magnesium (HBSS^-^, Gibco; Waltham, MA, USA). The bone marrow suspension was mechanically dispersed, centrifuged (1300 rpm, 6 min, 4°C) and subjected to brief hypotonic lysis (0.2 and 1.6% NaCl) to remove erythrocytes. Cells were then separated using a three-layer Percoll gradient (52%, 69%, 78%), and the neutrophil fraction was collected, washed, and resuspended in Hanks’ Balanced Salt Solution with calcium and magnesium (HBSS^+^; Gibco). Cell count, viability, and purity were assessed by hemocytometry using Trypan blue and Türk’s solution and by flow cytometry (Ly6G^+^ cells), yielding ~99% viable and approximately 90% pure neutrophils (98-99% based on morphology).

### Treatment with dimethyl fumarate

Bone marrow derived neutrophils were treated for 1 h with dimethyl fumarate (DMF) at a concentration of 25 µM (Merck, Darmstadt, Germany; resuspended in dimethyl sulfoxide; DMSO) prior to LPS stimulation. Further dilutions of DMF were preprepared in HBSS^+^. The remaining control groups were treated with the equivalent volume of DMSO. DMSO alone did not influence any of the measured parameters; therefore, these data are not shown.

### LPS stimulation

Neutrophils were stimulated for 3 or 6 h with lipopolysaccharide (LPS, *Pseudomonas aeruginosa serotype* 10.22; Merck) at a final well concentration of 50 µg/mL.

### Mitochondrial dynamics: induction or inhibition

In some experiments, Mdivi-1 (20 µM; MedChemExpress, NJ, USA), which promotes mitochondrial fusion, or CCCP (Carbonyl cyanide 3-chlorophenylhydrazone; 5 µM; MedChemExpress), which induces mitochondrial fragmentation/fission, was used to alter mitochondrial dynamics. The experiments were undertaken in either of two following regimes: addition of Mdivi-1/CCCP 1 h prior to LPS (Mdivi-1/CCCP (1 h)→LPS), addition of Mdivi-1/CCCP prior to DMF, then followed by LPS (Mdivi-1/CCCP (30 min)→DMF (30 min)→LPS). Expected mitochondrial morphology to be induced by Mdivi-1 and CCCP was confirmed by fluorescent and transmission microscopy under “resting” and “activated” conditions (**Supplementary Figure 3**) as described below.

### Inhibitors of metabolic pathways/regulated cell death

In some experiments, inhibitors of different metabolic pathways and regulated cell death (RCD) were applied in either of three following regimes: addition of inhibitor 1 h prior to LPS (inhibitor (1 h)→LPS), addition of inhibitor after DMF/CCCP and then addition of LPS (DMF/CCCP (30 min)→inhibitor (30 min)→LPS), addition of inhibitor prior to DMF and then LPS stimulation (inhibitor (30 min)→DMF (30 min)→LPS). The following inhibitors were used: nuclear factor erythroid 2-related factor 2 (NRF2) inhibitor – ML385 (10 µM, MedChemExpress), formyl peptide receptor 2 (Fpr2) antagonist - WRW4 (10 µM, MedChemExpress), NADPH oxidase (NOX) inhibitor - Diphenyleneiodonium chloride (DPI, 10 µM, Merck), necroptosis inhibitor - Necrostatin-1 (50 µM, Torcis Bioscence, Bristol, UK), pyroptosis inhibitor - Disulfiram (30 µM, MedChemExpress) and mitochondrial complex I inhibitor - Rotenone (5 µM, Sigma-Aldrich, Saint Louis, MO, USA).

### Neutrophil viability: PrestoBlue^®^ assay

Neutrophil viability was assessed with PrestoBlue^®^ assay (Invitrogen, Waltham, MA, USA). After 5.5 h of incubation with DMF stimulated with LPS (DMF (1 h)→LPS) or solo, 10 µL (1:10 ratio) of PrestoBlue^®^ reagent was added to each well containing 100 µL of treated cell suspension; then the plate was placed in an incubator for 30 min at 37°C. Fluorescence was measured in TECAN Infinite M Plex plate reader with 535 nm excitation and 595 nm emission wavelengths. Data is expressed as fluorescence arbitrary units (AU).

### Immunocytochemical staining of NETs

After 6 h of incubation, neutrophils were fixed in 4% paraformaldehyde (PFA, Alfa Aesar, Haverhill, MA, USA), and washed in PBS (Gibco). Subsequently, extracellular staining for citrullinated histone H3 (citH3), neutrophil elastase (NE), and extracellular DNA (extDNA) was performed. The coverglasses were washed twice with PBS and incubated in 3% bovine serum albumin (BSA; Merck) for 45 min at room temperature (RT). Next, rabbit polyclonal anti-histone H3 (citrulline R2+R8+R17) antibody (Abcam, Cambridge, UK) or rabbit monoclonal neutrophil elastase (E8U3X) antibody (Cell Signaling Technology, Danvers, MA, USA), both diluted 1:200 in 1% BSA were added to the fixed cells for overnight (at 4°C). After primary antibody incubation, the coverslips were washed twice with PBS and incubated for 1 h, RT in the dark with Cy3-conjugated goat anti-rabbit IgG (H+L) or Alexa Fluor^®^ 647 AffiniPure^®^ goat anti-rabbit IgG (H+L) secondary antibodies (1:300 in 1% BSA; Jackson ImmunoResearch Laboratories, Baltimore Pike, OH, USA). Next, the coverslips were washed with PBS and stained with Sytox Green (1:1000; Invitrogen) for 5 min to visualize extDNA. After a final PBS wash, the samples were mounted onto slides using Vectashield mounting medium (Vector Laboratories, Burlingame, CA, USA). To validate the use of Sytox Green as a DNA marker in fixed neutrophils, parallel staining with Hoechst and Sytox Green was performed following fixation. Under these conditions, both dyes produced fully overlapping nuclear signals in control and LPS-stimulated cells, confirming that Sytox Green reliably labels nuclear DNA in fixed neutrophils. Brightfield and phase-contrast imaging were used to verify cellular morphology.

### Visualization of mitochondrial dynamics

To visualize mitochondrial dynamics and localization, cell-permeant MitoTracker™ Green FM (Invitrogen, Waltham, MA, USA) probes were used. Neutrophils were cultured on glass coverslips and following stimulation with DMF and mitochondrial dynamics modulators, as well as treatment with LPS, a pre-warmed (37°C) MitoTracker™ Green FM staining solution (20 nM) was added for 40 min. After incubation (37°C), the coverslips were washed with HBSS^+^, and Hoechst 33342 dye (1:500; Invitrogen, Waltham, MA, USA) was applied to stain cell nuclei. Finally, the coverslips were mounted onto microscope slides using Vectashield mounting medium.

### Mitochondrial mass measurement by flow cytometry

Mitochondrial mass was assessed using MitoTracker™ Green FM, which stains active mitochondria in live cells. Neutrophils were stimulated with DMF/Mdivi-1/CCCP/LPS (as described above) and cultured (6 x 10^5^/600 µL) in flow cytometry tubes. After 3 h of LPS treatment, neutrophils were centrifuged (1500 rpm, 6 min, 4°C) and resuspended in pre-warmed (37°C) MitoTracker™ Green FM staining solution. Following incubation (40 min, 37°C), the cells were centrifuged again, washed, and resuspended in fresh HBSS^+^. Samples were then analyzed using Cytek Aurora spectral flow cytometer (Cytek Biosciences, CA, USA; app. 12–000 cells/per sample). The assessment of mitochondrial mass was performed using Kaluza Analysis 2.3 software (Beckman Coulter, Bread, CA, USA).

### Immunocytochemical staining of Nrf2, phospho-DRP-1 (Ser616), MFN1, MFN2, OPA1 and ZBP-1

Immunostaining of nuclear factor erythroid 2-related factor 2 (Nrf2), phosphorylated Dynamin-related protein 1 (DRP1) at Serine 616, mitofusin-1 (MFN1), mitofusin-2 (MFN2), optic atrophy protein-1 (OPA1) and Z-DNA-binding protein 1 (ZBP-1) was performed. Neutrophils seeded on coverslides were permeabilized by bathing in TBS (0.25% Triton X-100, 3.5% Na_2_HPO_4_×12H_2_O, 0.42% Na_2_HPO_4_×1H_2_O, 0.025% BSA, 5% NaCl, dH_2_O) or 0.1% Triton X-100 and blocked by incubation with 3% BSA (45 min). Subsequently, overnight incubation with rabbit monoclonal Nrf2 antibody (diluted 1:200 in 1% BSA; Cell Signaling Technology), rabbit polyclonal Phospho-DRP1 (Ser616) antibody (diluted 1:100 in 1% BSA; Invitrogen), rabbit polyclonal MFN1 (diluted 1:200 in 1% BSA; Proteintech, IL, USA), rabbit polyclonal MFN2 (diluted 1:200 in 1% BSA; Proteintech), rabbit polyclonal OPA1 (diluted 1:200 in 1% BSA; Proteintech) or rabbit polyclonal ZBP-1 antibody (diluted 1:50 in 1% BSA; Proteintech) was performed (4°C). After 18 h, the coverslips were washed twice with PBS and incubated for 1 h with Cy3-conjugated goat anti-rabbit IgG (H+L) secondary antibody (1:300 in 1% BSA) that matched all primary antibodies. The slips were then washed with PBS and stained with Sytox Green (1:1000) to visualize cell nucleus. Finally, the slides were rewashed in PBS and mounted using Vectashield medium.

### Detection of Annexin A1

Levels of Annexin A1 (ANXA1) in neutrophil supernatants were measured by Mouse Annexin A1 (Anxa1) ELISA Kit (Assay Gennie, Dublin, Irland). After stimulation of neutrophils with DMF or ML385 (1 h) alone, as well as a combination of DMF (30 min)→ML385 (30 min) (as described above), the supernatants were collected following LPS treatment. The ANXA1 assay was carried out as indicated by the manufacturer.

### Detection of apoptosis and necrosis: the Annexin V/PI assay

Annexin V-FITC Apoptosis Detection Kit I (BD Biosciences, San Diego, California, USA) was used to assess the percentage of apoptotic and necrotic neutrophils following CCCP treatment. Neutrophils were cultured in flow cytometry tubes and then washed with HBSS^+^ by centrifugation at 1500 rpm, 6 min, 4°C. Then, 100 μL of binding buffer, annexin V, and propidium iodide (PI) were added to the cell suspension. Samples were incubated at RT in the dark for 15 min. Subsequently, 400 μL of binding buffer was added to each tube, and the samples were placed on ice until measurement. For analysis, Navios flow cytometer (Beckman Coulter, Bread, CA, USA) was used (app. 12–000 cells/per sample). The assessment of apoptotic/necrotic cells after CCCP treatment was performed using Kaluza Analysis 2.3 software.

### Detection of Caspase 3/7 activity

A CellEvent™ Caspase 3/7 Green Flow Cytometry Assay Kit (Invitrogen) was used to detect activated caspases 3/7 in the neutrophils treated with DMF or CCCP cultured in flow cytometry tubes. Cells were centrifuged (1500 rpm, 6 min, 4°C) and their pellets were resuspended in 500 µL of HBSS^+^. Next, 0.5 μL/per sample of CellEvent^®^ Caspase-3/7Green Detection Reagent was added (30 min, 37°C). The fluorescent signal was measured immediately using the Navios flow cytometer (app. 12–000 cells/per sample). The percentage of neutrophils with active caspase 3/7 was determined using Kaluza Analysis 2.3 software.

### Dead cells/vital NETosis labeling with Live-or-Dye™

Live-or-Dye™ Fixable Viability Staining Kit (Live-or-Dye™ 568/583; Biotium, Kelowna, Canada) was employed to label non-viable neutrophils after CCCP treatment and to assess whether the observed NET release was occurring *via* the vital pathway. For flow cytometric analysis: Neutrophils incubated in flow cytometry tubes and treated with CCCP and/or LPS were centrifuged and washed with 0.5 mL of HBSS^+^. Next, cells were centrifuged one more time, resuspended in 500 µL of fresh HBSS^+^ and 0.5 µL of fixable Live-or-Dye™ was added to each tube (1:1000) – incubation time: 30 min RT, protected from light. The fluorescent signal was measured immediately using the Navios flow cytometer (12–000 cells/sample). The assessment of dead cells was performed using Kaluza Analysis 2.3 software. For confocal microscopic analysis: Staining of NETs with Live-or-Dye™ 568/583 after treatment with CCCP or/and LPS was performed on neutrophils seeded on coverslips using the same procedure as that applied for preparing samples for flow cytometry. Immediately after incubation, the cells were fixed in 4% paraformaldehyde and washed in PBS. Subsequently, the extracellular staining for citH3 and extDNA (indicative of NETs) was performed as described above.

### FDA-based assessment of metabolic activity

Fluorescein diacetate (FDA) (Invitrogen) was used to measure both enzymatic activity and cell-membrane integrity in neutrophils treated with CCCP and/or LPS. Cells were centrifuged, resuspended in 500 µL of warm (37°C) HBSS^+^ and 5 µL of FDA was added to each tube (15 min at 37°C). Next, 2.5 μL of PI staining solution was added to the cell suspension for 5 min. Fluorescence was immediately measured using the Navios flow cytometer (12–000 cells/sample). The assessment of metabolically active cells was performed using Kaluza Analysis 2.3 software.

### Assessment of mitochondrial membrane potential by TMRE

The tetramethylrhodamine ethyl ester perchlorate (TMRE; Merck) assay was used to analyze the mitochondrial membrane potential (Δψm). To the cells previously treated with CCCP, DMF, and/or LPS, 100 μL of TMRE solution (100 nM) was added, and the cells were incubated for 30 min at 37°C. To stop the reaction, 500 µL of HBSS^+^ buffer was added. Hoechst 33342 dye (1:500; 5 min) was applied to stain cell nuclei and the Δψm was examined under the microscope.

### Fluorescent microscopy analysis

After staining with TMRE or Sytox Green/Hoechst 33342 to show overlapping co-localization of two nuclear dyes, a fluorescent signal, brightfield and phase contrast were detected with a ZEISS Axiovert 5 inverted microscope equipped with camera Axiocam 208 color and fluorescence LED illumination Colibri 3. Fluorescent signal was detected in three channels DAPI (390/40 nm Ex, 450/40 nm Em), GFP (470/40 nm Ex, 525/50 nm Em) and Cy3 (550/25 nm Ex, 605/70 nm Em) using ZEISS ZEN 3.12 Imaging software (Zeiss, Oberkochen, Germany).

### Confocal microscopy analysis

After immunocytochemical and MitoTracker™ Green FM staining, a fluorescent signal was detected with a ZEISS Axio Examiner.Z1 upright microscope equipped with a metal halide light source (AMH-200-F6S; Andor, Oxford Instruments, Abingdon, UK) and confocal spinning disk device DSD2 (Andor, Oxford Instruments, Abingdon, UK) with ZEISS EC Plan‐NEOFLUAR 20×/0.5 air objective) in four channels: GFP (482/18 nm Ex, 525/45 nm Em); RFP (561/54 nm Ex, 609/54 nm Em); DAPI (390/40 nm Ex, 452/45 nm Em); Cy5 (640/14 nm Ex, 676/29 nm Em) using IQ 3. 6. 1. Live Cell Imaging software (Andor, Oxford Instruments, Abingdon, UK). The immunocytochemical images were analyzed using ImageJ v1.53a (National Institutes of Health, USA).

### Transmission electron microscopy analysis of neutrophil death

Neutrophils were cultured in flow cytometry tubes (6 x 10^5^/600 µL) in three experimental groups: an unstimulated control group (CTR), an LPS-stimulated group (LPS), and a group treated with CCCP followed by LPS stimulation (CCCP (1 h)→LPS). After 3 h of incubation, neutrophils were centrifuged (15–000 rpm, 6 min, 4°C), and the resulting pellets were resuspended in a fixative (2.5% glutaraldehyde in 0.1 M cacodylic buffer) and incubated for 24 h at 4°C. Then, samples were dehydrated in graded ethanol 50%, 70%, 96% and 100%. After incubation in propylene oxide, samples were embedded in Poly/Bed^®^ 812 epoxy resin at 68°C. Next, ultrathin sections, about ~70 nm thickness, were cut using microtome Leica UC7, placed on 300-mesh Formvar/Carbon grids and contrasted using uranyl acetate and lead citrate. Visualization of neutrophils was performed with a JEOL JEM 2100HT (Jeol Ltd, Tokyo, Japan) transmission electron microscope TEM that was used at an accelerating voltage of 80 kV. Images were taken by using 4 k × 4 k camera (TVIPS) equipped with EMMENU software ver. 4.0.9.

### Statistics

All data are presented as mean ± SD. Normality of distribution was assessed prior to statistical testing. For normality distributed data, comparisons were performed using one-way ANOVA or two-way ANOVA followed by Bonferroni *post hoc* analysis. For data that did not meet normality assumptions, the non-parametric equivalent Kruskal-Wallis test with Dunn’s *post hoc* test were used. Any two means that do not share the same letter are significantly different according to these analyses. Statistical significance was set at p < 0.05.

## Results

### Fumarate, a TCA intermediate metabolite, inhibits NETs

**Figure 1:**
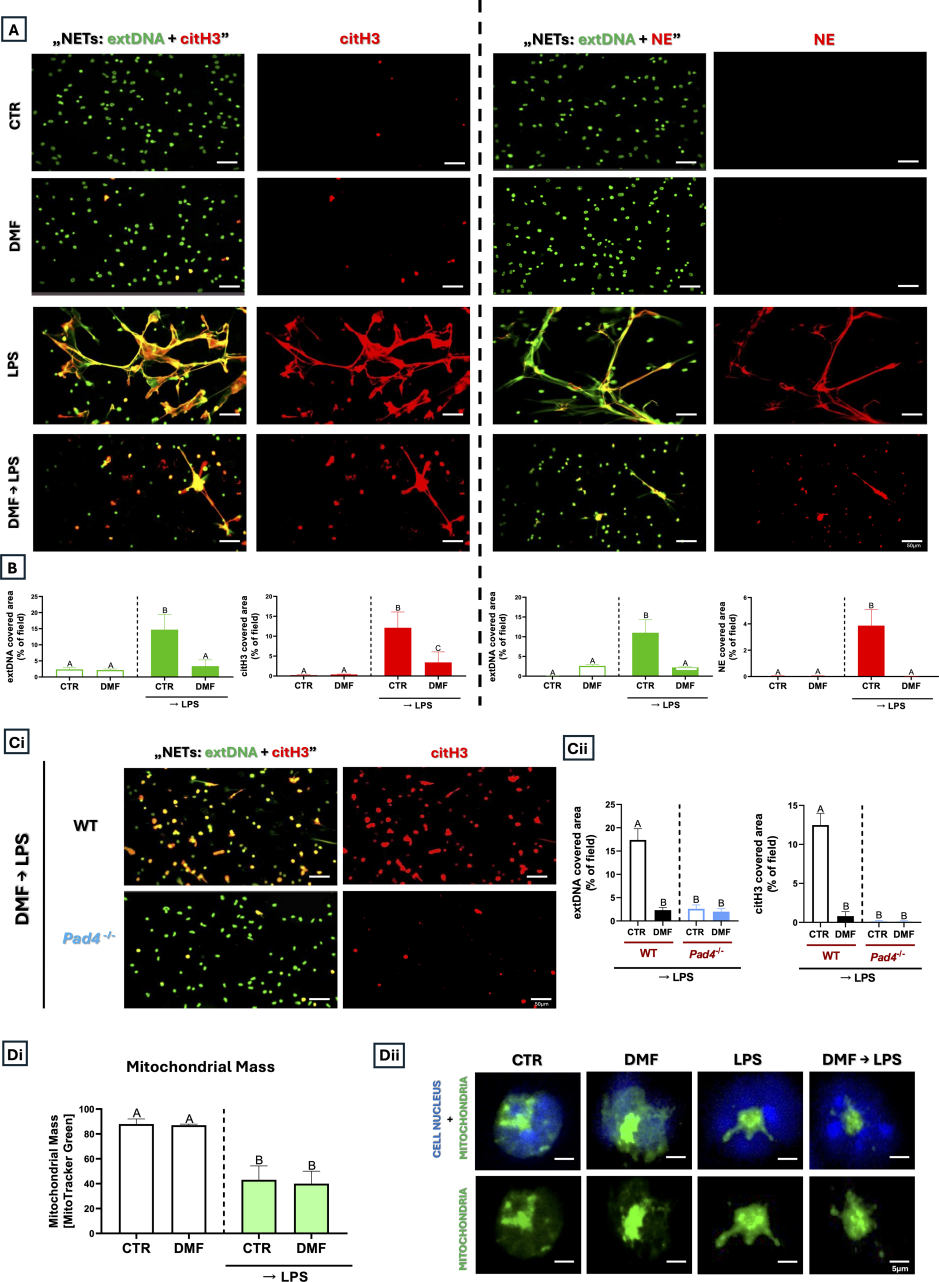
Impact of the dimethyl fumarate, a Krebs cycle metabolite, on the ability to form NETs by neutrophils isolated from C57BL/6J wild-type and peptidylarginine deiminase 4 KO (*Pad4*^-/-^*)* mice. Neutrophils were left unstimulated (CTR), stimulated with LPS (50 µg/mL), pre-treated with DMF (25 µM, 1 h), or pre-treated with DMF followed by 6 h of LPS stimulation (DMF→LPS). Data presented in A-B and D was obtained in C57BL/6J (WT) mice. Representative images show NETs formed in response to LPS in the presence of DMF. **(A)** extDNA is shown in green, with citH3 (left panel) or NE (right panel) shown in red. Merged images indicate co-localization of NET components **(B)** Quantification of NET formation: area covered by the extDNA, citH3 and NE signal. **(Ci)** Representative images of NETs formed upon the DMF treatment followed by LPS stimulation (DMF→LPS) on the neutrophils isolated from WT or *Pad4*^-/-^ mice and **(Cii)** area of extDNA and citH3 signal upon the above treatments. **(Di)** Measurement of Mitochondrial Mass with MitoTracker Green in flow cytometry upon the above treatments. (Dii) Representative images of untreated (CTR) neutrophils or treated with DMF, LPS or DMF and LPS (DMF→LPS) for 3 h Mitochondria (MitoTracker) are shown in green while cell nucleus (Hoechst) in blue. The results are expressed as the mean values ± SD; n≥3. Values significantly different between the groups (p < 0.05) according to one-way ANOVA, two-way ANOVA (*post hoc* Bonferroni test) or Kruskal-Wallis test with Dunn’s *post hoc* are designated by letters, where the same letter indicates no differences between groups (different letters indicate statistical differences). Explanation of abbreviations: extDNA, extracellular DNA; citH3, citrullinated histone H3; NE, neutrophil elastase.

To induce NETs and test the impact of fumarate on their formation, neutrophils were pretreated with methyl ester of fumaric acid - dimethyl fumarate (DMF), and then stimulated with a potent biological NET inducer, lipopolysaccharide (LPS). LPS itself induced the release of NETs as confirmed by the co-localization of extracellular DNA (extDNA) with citrullinated histones H3 (citH3, **Figures 1A, B** left panel) and granule-derived neutrophil elastase (NE) (**Figures 1A, B** right panel). Significantly, pre-treatment of neutrophils with DMF for 1 h before LPS stimulation strongly inhibited NET formation (**Figures 1A, B**). Peptidylarginine deiminase 4 (PAD4) is a key enzyme responsible for histone citrullination during NET formation (37), and its genetic depletion (P*ad4*^-/-^; KO) results in severely impaired or absent NETs (**Supplementary Figure 2A**). Accordingly, we tested the effects of LPS and DMF on neutrophils from wild-type (WT) and KO mice to determine whether we could detect NETs and whether the absence of histone citrullination alters the DMF-induced response. However, whereas we confirmed PAD4-dependency of NET formation upon LPS stimulation, co-administration of DMF had the same effect in KO as in WT animals (**Figure 1Ci, Cii**). Validation experiments demonstrated that in fixed neutrophils Sytox Green co-localizes with Hoechst nuclear staining (**Supplementary Figure 1**), confirming its suitability as a DNA marker in this setting.

### Fumarate causes fusion of mitochondria

**Figure 2:**
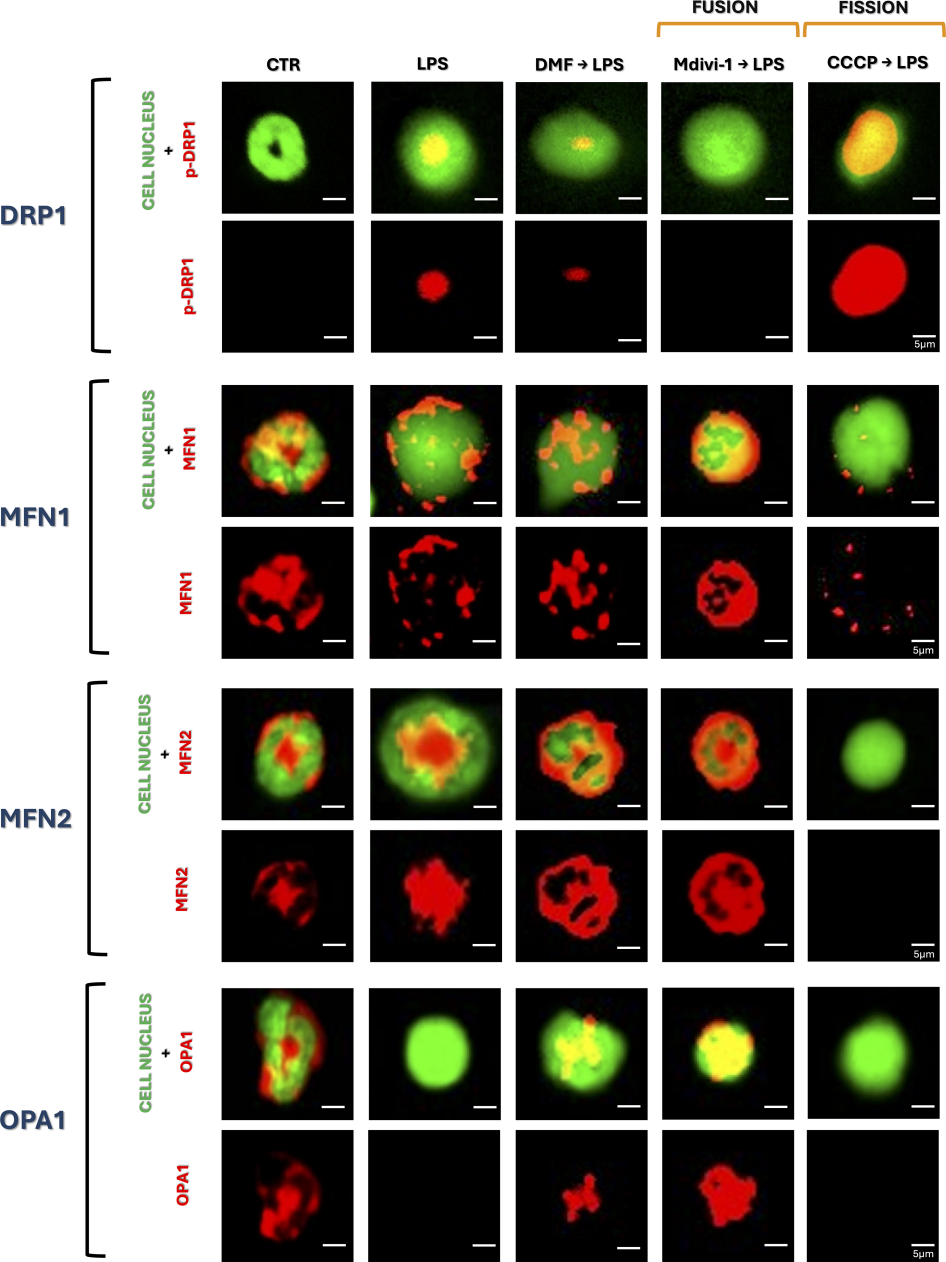
Mitochondria dynamics, their fusion or fission/fragmentation shown by expression of p-DRP1, MFN1, MFN2 and OPA1. Neutrophils isolated from mice were left unstimulated (CTR) or stimulated with LPS (50 µg/mL). Where indicated cells were pre-treated with Mdivi-1 (20 µM), CCCP (5 µM), or DMF (25 µM). Representative images of p-DRP1 (Ser616; first panel), MFN1 (second panel), MFN2 (third panel) and OPA1 (fourth panel) shown in red and nucleus in green (Sytox green) Explanation of abbreviations: p-DRP1, dynamin-related protein 1 phosphorylated form on Ser616; MFN1, mitofusin-1; MFN2, mitofusin-2; OPA1, optic atrophy protein-1.

**Figure 3:**
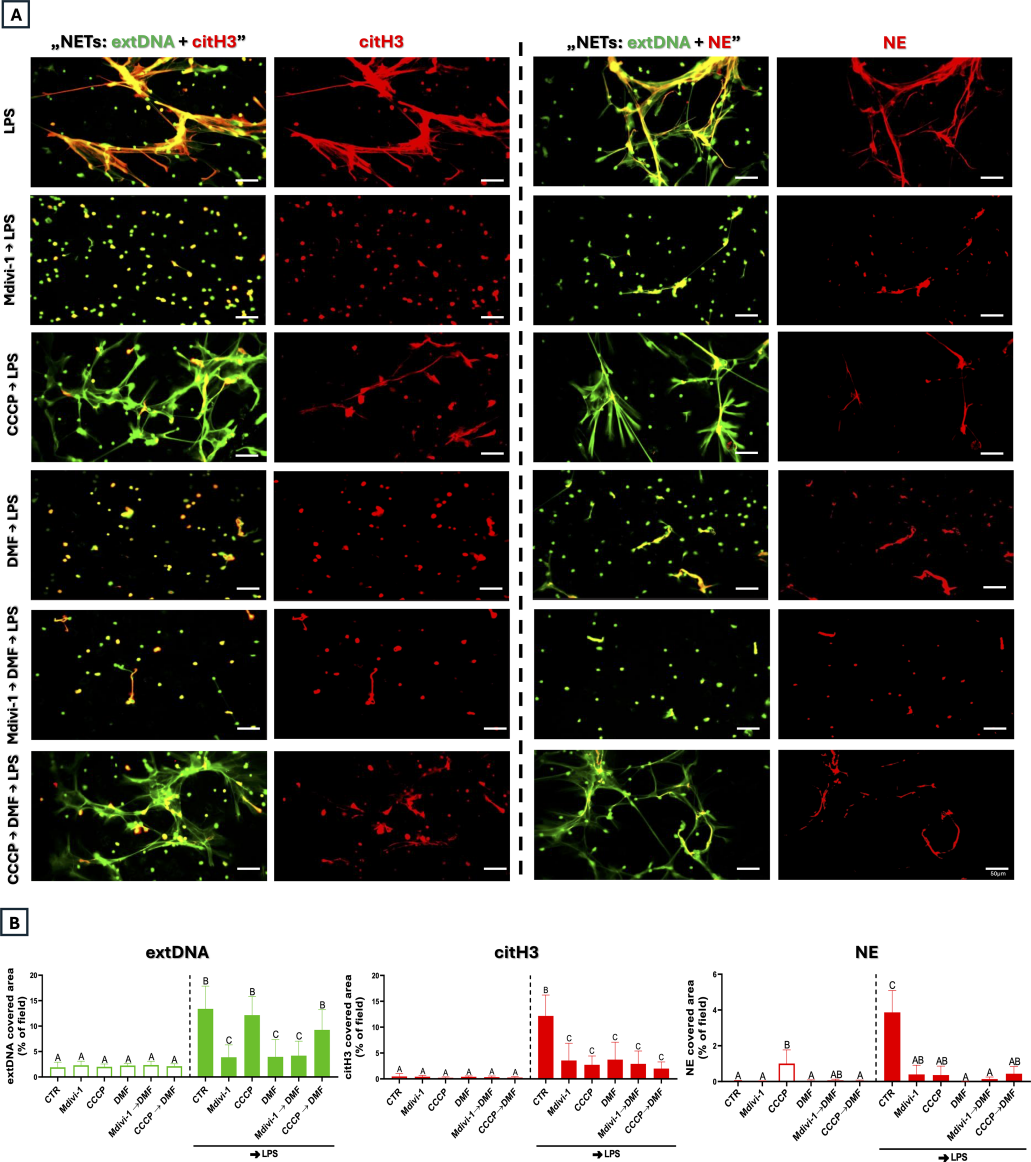
Mdivi-1 (fusion) and CCCP (fission/fragmentation) effect on NET release. Neutrophils isolated from mice were stimulated with LPS (50 µg/mL). Where indicated, cells were pre-treated with Mdivi-1 (20 µM), CCCP (5 µM), or DMF (25 µM) or sequentially treated with Mdivi-1 or CCCP followed by DMF to LPS stimulation (Mdivi-1/CCCP→DMF→LPS). Representative images of **(A)** NETs: extracellular DNA (extDNA) in green, citrullinated histone H3 (citH3; left panel) and neutrophil elastase (NE; right panel) in red. **(B)** Quantification of NET formation: area covered by the extDNA, citH3 and NE signal. The results are expressed as the mean values ± SD; n≥3. Values significantly different between the groups (p < 0.05) according to one-way ANOVA (*post hoc* Bonferroni test) are designated by letters, where the same letter indicates no differences between groups (different letters indicate statistical differences).

To identify the mechanism of DMF-mediated inhibition of NETs, we first focused on mitochondria. Especially considering that fumarate is an intermediate metabolite of the tricarboxylic acid cycle (TCA) occurring in the mitochondrial matrix (22). LPS stimulation reduced mitochondrial mass relative to untreated cells, and DMF did not alter this phenotype (**Figure 1Di, Dii**). To determine the nature of mitochondrial dynamics following LPS and DMF treatment, we examined the activity of the dynamin-related protein 1 (DRP1) and, specifically, its phosphorylated form (p-DRP1; Ser616), together with key mitochondrial fusion markers - mitofusin-1 (MFN1), mitofusin-2 (MFN2), and optic atrophy protein-1 (OPA1). Phosphorylation of DRP1 proves the occurrence of mitochondrial fragmentation/fission (25), while its absence confirms the occurrence of mitochondrial fusion (**Figure 2**). As positive and negative controls, we used Mdivi-1 (fusion) and CCCP (fragmentation/fission), respectively. The compounds induced the expected mitochondrial changes, as confirmed by confocal microscopy under “resting” (no LPS) and “activated” (LPS) conditions (**Supplementary Figure 3A**). The more detailed analysis showed an almost complete absence of DRP1 phosphorylation after stimulation of neutrophils with DMF, which suggests a shift towards a mitochondrial fusion occurs even more strongly upon DMF than LPS itself. Consistently, MFN1, MFN2, and OPA1 displayed a more continuous and interconnected staining pattern similar to that seen with Mdivi-1. In contrast, CCCP induced a punctate MFN1 pattern characteristic of mitochondrial fragmentation, while MFN2 and OPA1 signals were absent. LPS stimulation also resulted in altered MFN1/MFN2 distribution and diminished OPA1 signal, suggesting disruption of inner mitochondrial membrane organization. (**Figure 2**). In addition, a cytometric analysis of mitochondrial mass showed its decrease after LPS and Mdivi-1→LPS treatments (similarly to DMF), and this effect was much weaker in the case of mitochondrial fragmentation (CCCP→LPS), supporting a fusion-dominant phenotype in DMF-treated cells (**Supplementary Figure 3B**). Given that mitochondrial dynamics can affect neutrophil activation, we examined how Mdivi-1 and CCCP treatment affect NET formation. The analysis showed that mitochondrial fusion (Mdivi-1) completely inhibited NET formation (all three components tested), the effect corresponding to that of DMF. When Mdivi-1 and DMF were added together, the result was the same. To further support the role of DRP1-dependent mitochondrial dynamics, we additionally tested dynasore as an alternative dynamin inhibitor. Dynasore similarly reduced p-DRP1 (Ser616) levels and suppressed NET-associated extDNA and citH3 release (data not shown). In contrast, mitochondrial fragmentation did not alter extDNA release, although it did inhibit the release of NET proteins, citH3, and NE. Cell pretreatment with CCCP before DMF produced the same phenotype as CCCP alone, and thus was opposite to the action of DMF (**Figures 3A, B**). The observation on CCCP was puzzling; was it a partial inhibition of NETs (only its proteins), or was the source of extDNA different from NETs, e.g., resulting from cell death with a necrotic morphology? Before resolving this issue, we continued to investigate the mechanism of DMF action.

### DMF does not cause neutrophil death

**Figure 4:**
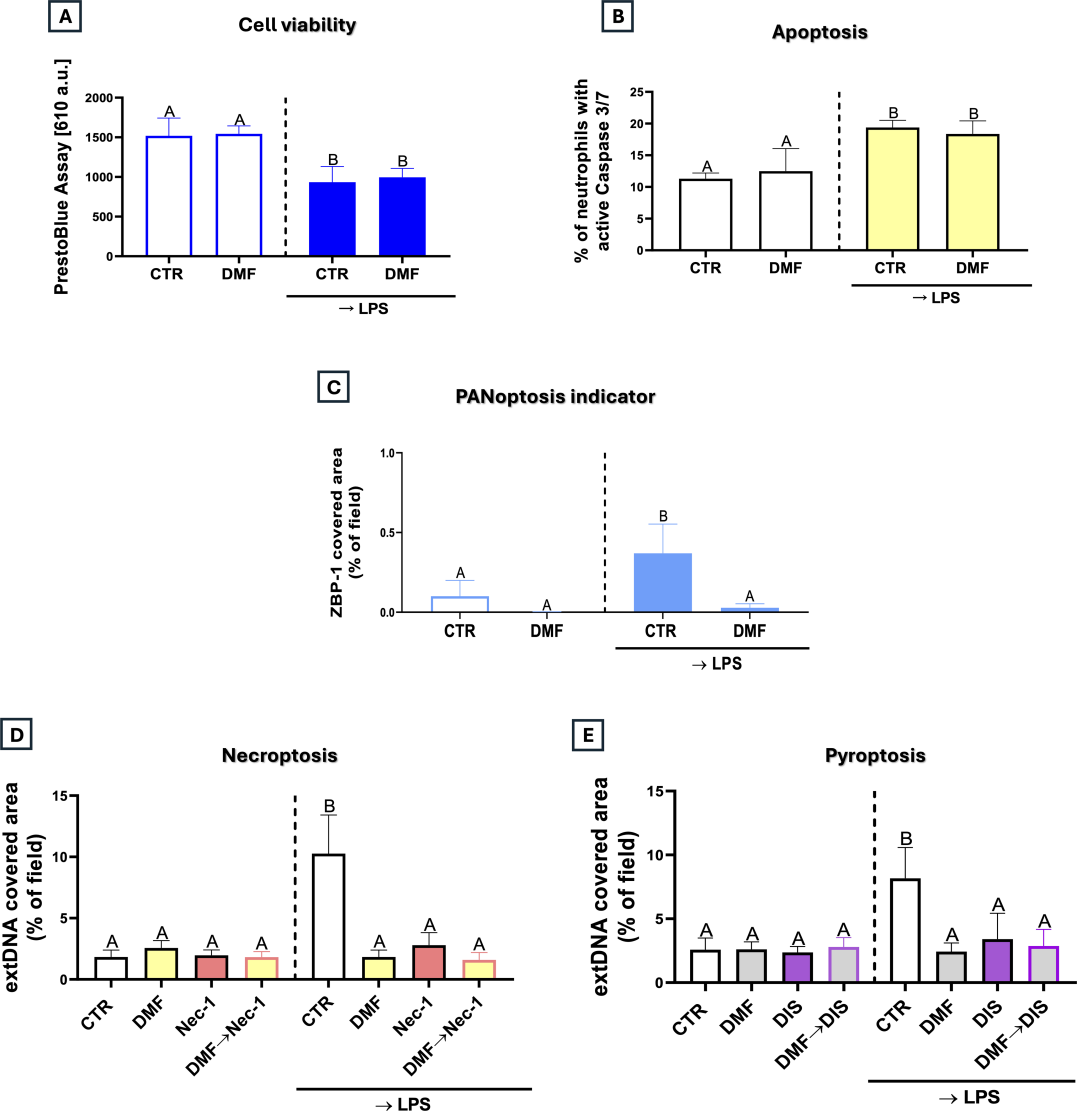
Impact of dimethyl fumarate on the regulated cell death of neutrophils. The analyses were performed with **(A)** PrestoBlue (cell viability; spectrophotometry), **(B)** Caspase 3/7 activity assay (apoptosis; flow cytometry) and **(C)** quantification of ZBP-1 (PANoptosis indicator; confocal microscopy) expression respectively. Neutrophils isolated from the bone marrow were treated with DMF (25 µM) for 1h or they were left unstimulated (CTR). Some of the cells were subsequently stimulated with lipopolysaccharide (LPS) at a concentration of 50 μg/mL. To determine necroptosis **(D)** and pyroptosis **(E)** extDNA was quantified after DMF, application of specific inhibitors: Nec-1, DIS treatment (1 h) or their combination. Additionally some cells were pretreated with DMF (DMF→Nec-1/DIS) incubated with or without LPS. The results are expressed as the mean values ± SD; n≥3. Values significantly different between the groups (p < 0.05) according to one-way ANOVA (*post hoc* Bonferroni test) are designated by letters, where the same letter indicates no differences between groups (different letters indicate statistical differences. Explanation of abbreviations: ZBP-1, Z-DNA-binding protein 1; extDNA, extracellular DNA; Nec-1, necrostatin-1; DIS, disulfiram.

The effect of DMF in decreasing NET formation could have been mediated by its cytotoxicity toward neutrophils. To evaluate this, we tested for neutrophil viability, and the test based on evaluation of mitochondrial activity (Presto blue) showed that DMF does not cause further decrease of cell viability than that induced by LPS (**Figure 4A**). To make sure that LPS is not masking any effect of DMF, we also verified particular cell death pathways: apoptosis and necroptosis, as well as PANoptosis, a newer concept in cell death, describing a single, integrated process where cells simultaneously activate features of three major cell death pathways (apoptosis, pyroptosis, and necroptosis) (38). Whereas we did not detect differences in apoptotic cell counts between cells stimulated with DMF→LPS and LPS alone, ZBP1 levels, indicative of PANoptosis (39), were even lower in the presence of DMF than in the absence of DMF (**Figures 4B, C**). To more closely assess the involvement of necroptosis or pyroptosis in the effects of DMF, we used inhibitors of these cell death pathways (necrostatin-1 and disulfiram, respectively). Still, they did not alter the inhibitory effect of DMF on NETs, confirming that the compound does not act by inducing cell death (**Figures 4D, E**). Moreover, although LPS decreased mitochondrial membrane potential, DMF restored it, further supporting its cell protective role (**Supplementary Figure 4**). Because pharmacological modulators of mitochondrial dynamics may secondarily affect mitochondrial respiration, we next asked whether direct inhibition of mitochondrial oxidative phosphorylation is sufficient to suppress NET formation. Importantly, pharmacological inhibition of mitochondrial complex I with rotenone did not reduce NET release (**Supplementary Figure 5**), indicating that disruption of mitochondrial OXPHOS *per se* is not sufficient to inhibit NETs. These findings further support the notion that the NET-inhibitory effects observed in our model are not driven by impaired mitochondrial respiration but rather by specific alterations in mitochondrial dynamics.

### Nrf2 and Annexin A1 are involved in DMF-dependent NETs inhibition

**Figure 5:**
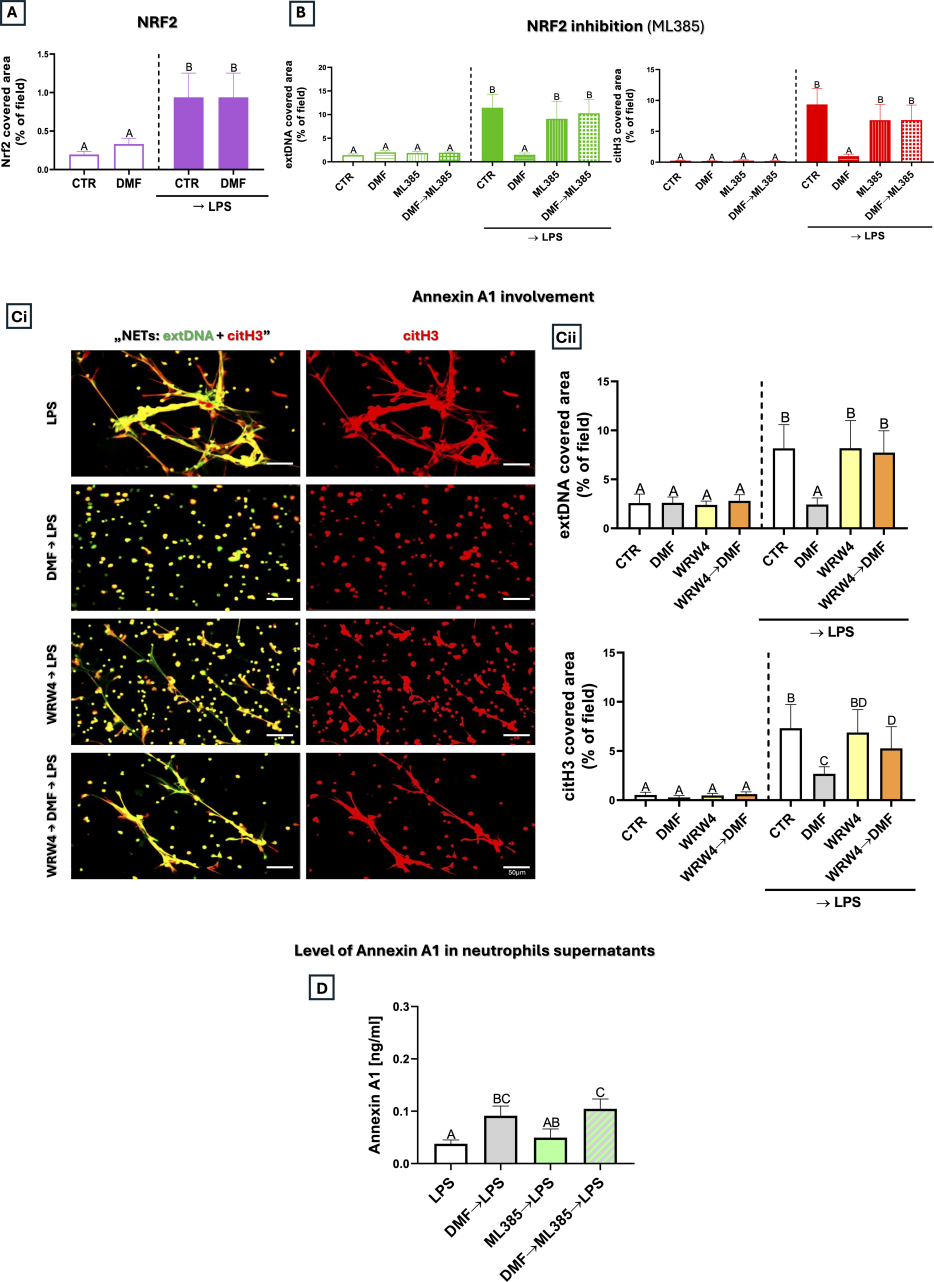
Expression of Nrf2 and ANXA1 in relation to NET release by neutrophils treated with dimethyl fumarate. Neutrophils isolated from mice were either pre-treated with DMF (25 µM), ML385 (Nrf2 inhibitor), WRW4 (Fpr2 antagonist) for 1 h or their combination: DMF→ML385, WRW4→DMF incubated with lipopolysaccharide (LPS) at a concentration of 50 μg/mL (for 6 h) or left unstimulated (CTR). **(A)** Quantification of Nrf2 expression and **(B)** NETs (extDNA and citH3) quantification after Nrf2 inhibition. **(Ci)** Representative images and **(Cii)** area of NETs formed upon the above treatments with WRW4 and DMF. ExtDNA is shown in green and citH3 is shown in red. To visualize co-localization of extDNA with citH3 expression, the images from each channel were overlaid. **(D)** Levels of ANXA1 were measured in neutrophils supernatants upon the above treatments with DMF and ML385 by ELISA. The results are expressed as the mean values ± SD; n≥3. Values significantly different between the groups (p < 0.05) according to one-way ANOVA (*post hoc* Bonferroni test) are designated by letters, where the same letter indicates no differences between groups (different letters indicate statistical differences). Explanation of abbreviations: Nrf2, nuclear factor erythroid 2-related factor 2; ANXA1, Annexin A1; extDNA, extracellular DNA; citH3, citrullinated histone H3.

To elucidate the mechanism of DMF action in more detail, and given that Nrf2 activation is known to play a role in the regulation of mitochondrial biogenesis (40), we sought to investigate how this pathway is affected by the DMF pre-treatment. As we showed previously (20), LPS alone leads to an increase in Nrf2 expression; however, pre-treatment with DMF did not further enhance this effect (**Figure 5A**). Importantly, we first added DMF and then the inhibitor, the strategy employed by us previously (20) when the pharmacological inhibition of downstream Nrf2 targets must be applied after the pathway activation (here: with DMF). When Nrf2 activity was pharmacologically inhibited following DMF treatment (DMF→ML 385), NET formation was restored to levels comparable to LPS stimulation alone (**Figure 5B**). This finding highlights Nrf2’s functional role in mediating DMF’s inhibitory effect on NETs. To further investigate this mechanism, we examined the involvement of Annexin A1 (ANXA1). ANXA1 is a protein involved in the resolution of inflammation and known to be regulated in an Nrf2-dependent manner (23). It was shown that DMF modifies KEAP1 cysteines, stabilizing and activating Nrf2, and this triggers a non-classical, ABCA1-dependent secretory pathway releasing a 33-kDa fragment of ANXA1 in macrophages (23). Silencing Nrf2 or ABCA1 abolishes this effect, and KEAP1 KO enhances it. Based on the above macrophage data (their setup: DMF→Nrf2→ANXA1 secretion), we hypothesized that in our system, DMF-induced ANXA1 might act on neutrophils *via* formyl peptide receptor 2 (Fpr2; the principal receptor for ANXA1 (41)) and thereby inhibit NETs. In line with this hypothesis, a pharmacological blockade of the Fpr2 receptor, before DMF treatment (WRW4→DMF), indeed restored NET formation, indicating that ANXA1 is involved in NET inhibition (**Figure 5Ci, Cii**). To verify whether DMF actually induces ANXA1 secretion by neutrophils, we measured its levels in the cell supernatants. We confirmed that in the presence of LPS, dimethyl fumarate increases ANXA1 secretion. In the next series of experiments, we evaluated the role of Nrf2 in regulating ANXA1 secretion. The Nrf2 inhibitor (ML385) did not statistically affect ANXA1 levels induced by LPS (these remained unchanged), however, combining the LPS stimulation with the DMF pre-treatment (DMF→ML385→LPS) resulted in secretion comparable to DMF→LPS (**Figure 5D**). These results indicate that inhibition of Nrf2 does not significantly affect DMF-induced ANXA1 secretion, suggesting that ANXA1 release occurs largely independently of Nrf2 signaling and may involve additional regulatory pathways. Nonetheless, the experiments with Nrf2 blockade (**Figure 5B**) clearly demonstrated that the activity of this transcription factor is necessary for DMF to inhibit NET formation even though it does not solely account for changes in ANXA1 secretion (**Figure 5D**). Collectively, these data suggest that DMF activates a multifaceted response in neutrophils that enhances ANXA1 secretion and engages Fpr2-dependent signaling, both of which contribute to the suppression of NET formation.

### CCCP-dependent extDNA release is not a NETosis

**Figure 6:**
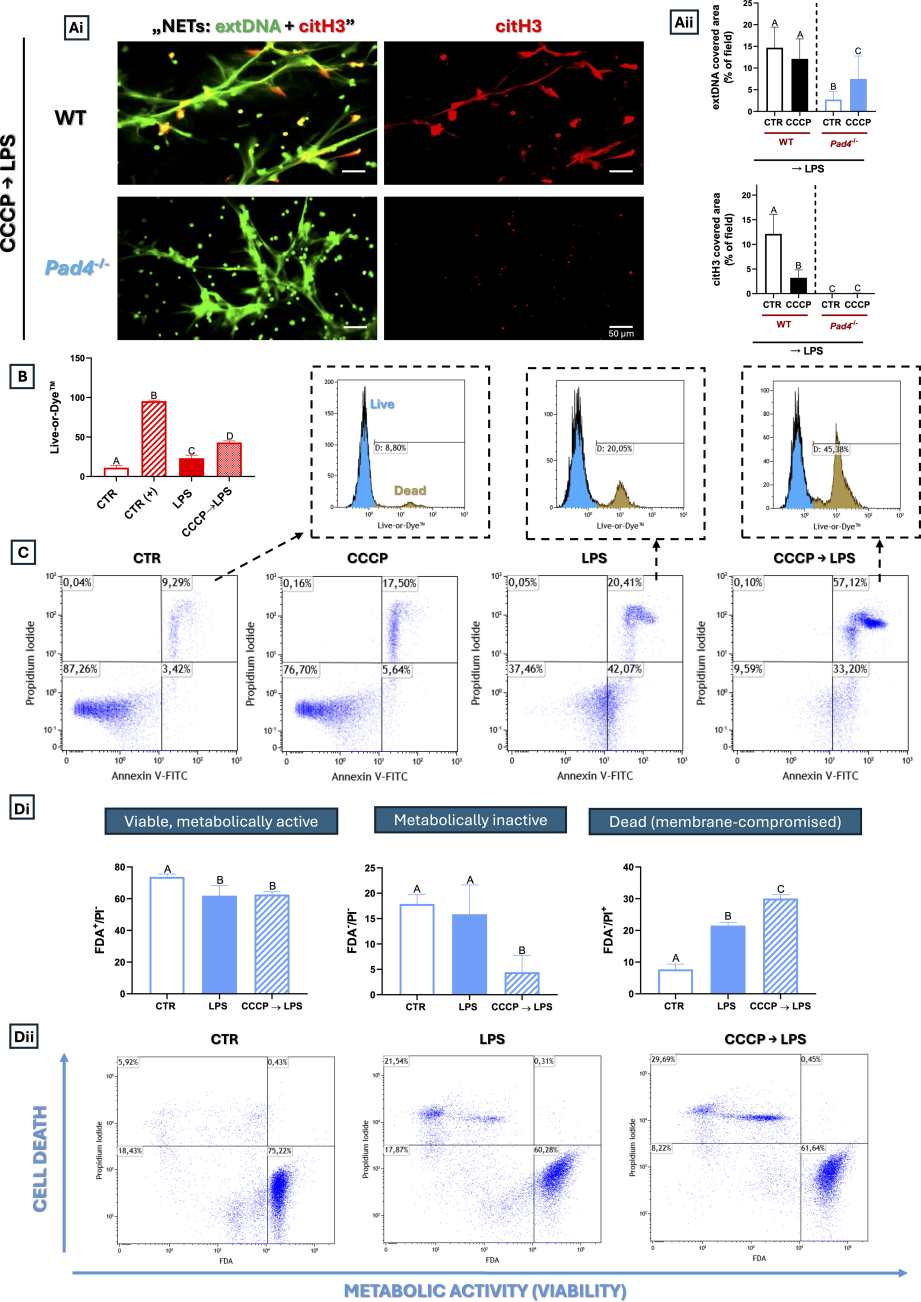
Effect of mitochondria fission/fragmentation on the ability to form NETs by neutrophils isolated from C57BL/6J wild-type and peptidylarginine deiminase 4 KO (*Pad4*^-/-^*)* mice and neutrophil cell death. Neutrophils from WT or *Pad4*^−/−^ mice were left unstimulated (CTR), treated with CCCP (5 µM, 1 h), stimulated with LPS (50 µg/mL, 6 h), or pre-treated with CCCP followed by LPS stimulation (CCCP→LPS). Representative images of NETs formed by LPS on the neutrophils isolated from WT or *Pad4*^-/-^ mice in the presence CCCP are shown in **(Ai)** extDNA is shown in green while citH3 in red. To visualize co-localization of NET components, the images from each channel were overlaid (NETs: extDNA + citH3). **(Aii)** Quantification of NET formation: area covered by the extDNA and citH3 signal. Data presented in B-D was obtained in C57BL/6J WT mice. The cytometric analyses upon above treatment were performed with **(B)** Live-or-Dye™ for dead cells labeling – EtOH was used as a positive control (CTR+) **(C)** Annexin V/PI Assay for apoptosis detection and **(Di, Dii)** FDA-based assessment of metabolic activity. The results are expressed as the mean values ± SD; n≥3. Values significantly different between the groups (p < 0.05) according to one-way ANOVA, two-way ANOVA (*post hoc* Bonferroni test) are designated by letters, where the same letter indicates no differences between groups (different letters indicate statistical differences. Explanation of abbreviations: extDNA, extracellular DNA; citH3, citrullinated histone H3.

Having established the mechanism by which DMF inhibits NETs, we returned to the effect of CCCP (**Figures 3A, B**). Could CCCP also act as a NET inhibitor or, on the contrary, its inducer? To determine whether the extracellular DNA (extDNA) released by cells after CCCP pretreatment is part of NETs or a morphological consequence of cell death with a necrotic morphology, we isolated neutrophils from *Pad4*^-/-^ mice and treated them with CCCP. Surprisingly, following LPS stimulation, PAD4-deficient neutrophils still released extracellular DNA (extDNA) after CCCP pretreatment (**Figure 6Ai, Aii**). This suggested release of non-canonical, PAD4-independent DNA structures, potentially unrelated to classical NETs. We hypothesized that CCCP might not inhibit NETs *per se*; instead, it induces cell death associated with cell rupture, leading to the release of cell DNA. Especially, CCCP reduced mitochondrial membrane potential (Δψm) much stronger than LPS (**Supplementary Figure 4**).

### CCCP-dependent mitochondrial fragmentation/fission drives neutrophil death

As the data collected thus far were indicative of cell death following CCCP pretreatment, we aimed to identify the mode of cell death. Flow cytometric evaluation of cell integrity by Live-or-Die™ dye, which enters dead cells with compromised membranes and covalently labels intracellular free amines, showed nearly a two-fold signal increase after application of CCCP in comparison to LPS alone (**Figure 6B**). This was further confirmed by microscopic analysis of the Live-or-Die™ signal upon the above treatment (**Supplementary Figure 6**). Aimed at apoptosis detection, Annexin V/PI staining revealed increased levels of early and late apoptotic cells with mitochondrial fragmentation in the CCCP→LPS treated cells (**Figure 6C**). We also performed FDA/PI staining indicative of necrosis, as intracellular esterases convert FDA into a fluorescent signal in metabolically active cells. The assay showed reduced metabolic activity/cell viability after CCCP→LPS treatment compared with LPS alone (**Figure 6Di, Dii**). Altogether, these data strongly indicate that mitochondrial fragmentation does not lead to canonical NET formation and is associated with loss of neutrophil viability and activation of apoptotic and necrotic pathways. Moreover, inhibition of NADPH oxidase (NOX) with DPI, which typically effectively blocks NETs (42), did not abolish extDNA release after CCCP, further confirming that the observed mechanism differs from classical NOX-dependent NETosis (**Supplementary Figure 7Ai, Aii**).

### CCCP is not involved in NET formation nor impacts the process

**Figure 7:**
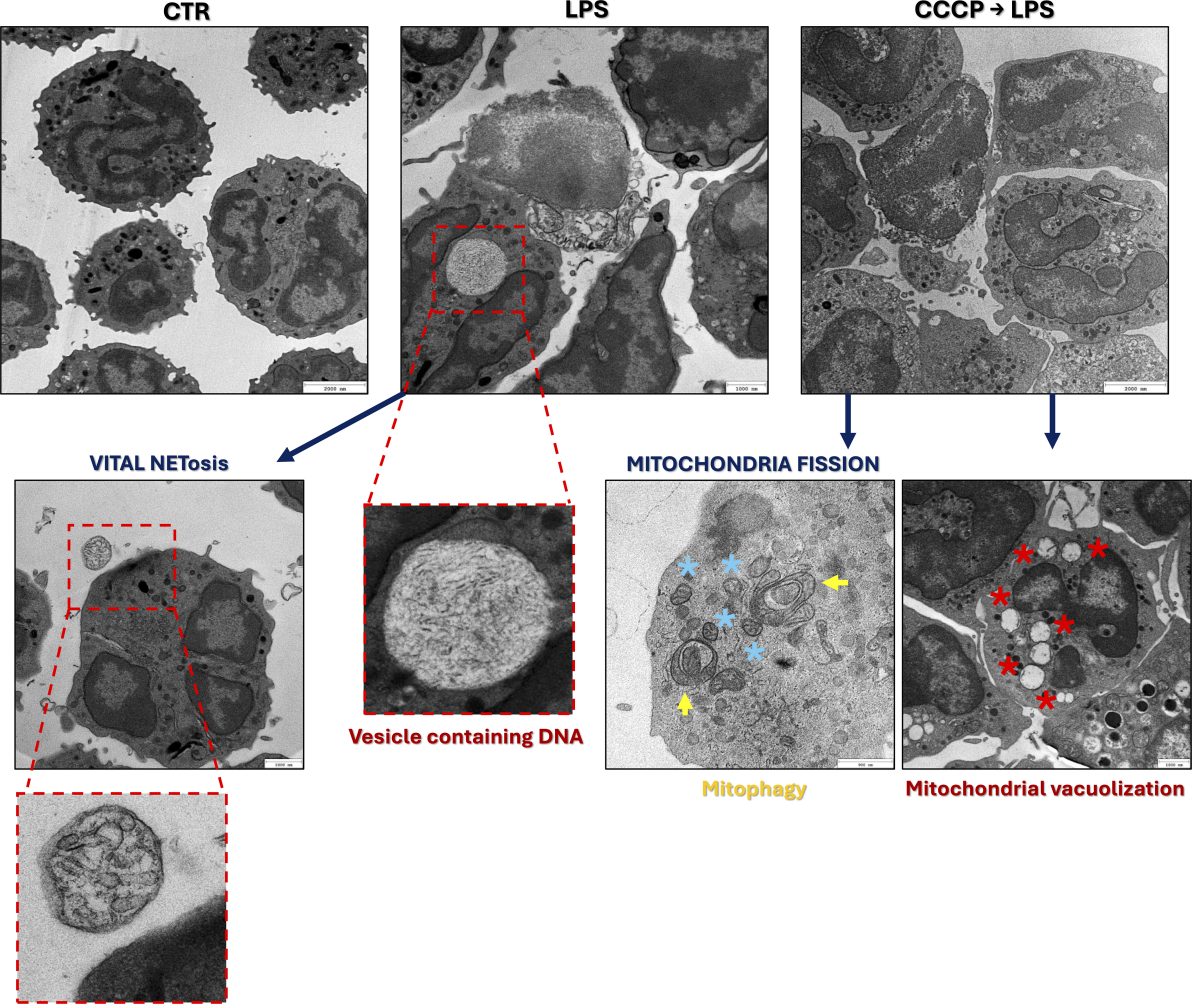
Ultrastructural analysis of neutrophils undergoing NET release in response to LPS and mitochondrial fission/fragmentation. Transmission electron microscopy (TEM) was used to visualize ultrastructural changes in neutrophils under the following conditions: unstimulated controls (CTR), stimulation with LPS (3 h), pre-treatment with CCCP (5 µM, 1 h) followed by LPS stimulation (CCCP→LPS). LPS stimulation induced features characteristic of vital NETosis, including presence of extracellular vesicle-like structure containing expelled DNA; an exemplary vesicle is highlighted with a red dashed line. Treatment with CCCP (CCCP→LPS) resulted in pronounced mitochondrial fragmentation (blue asterisks), mitophagic structures (yellow arrows), and vacuolization of mitochondria (red asterisks), indicating severe mitochondrial stress.

However, there was one more possibility left, CCCP could be inducing a unique NET-associated form of death which used to be called lethal NETosis (34). Data gathered thus far indicate that this process occurs approximately 6 h after LPS stimulation, as revealed by flow cytometric analysis and Live/Dead™ fluorescent staining. However, transmission electron microscopy (TEM) showed that 3 h after LPS stimulation, neutrophils displayed morphological features of vital NETosis (35, 43), i.e. that neutrophils remain viable after NET release, including vesicles containing decondensed chromatin (**Figure 7**). Conversely, CCCP-treated cells showed extensive ultrastructural damage, including mitochondrial fragmentation, widespread cell death, probable mitophagy, and pronounced mitochondrial vacuolization (**Figure 7**). Altogether, indicating cell death not related to NET release.

### PANoptosis is involved in CCCP-related extDNA release

**Figure 8:**
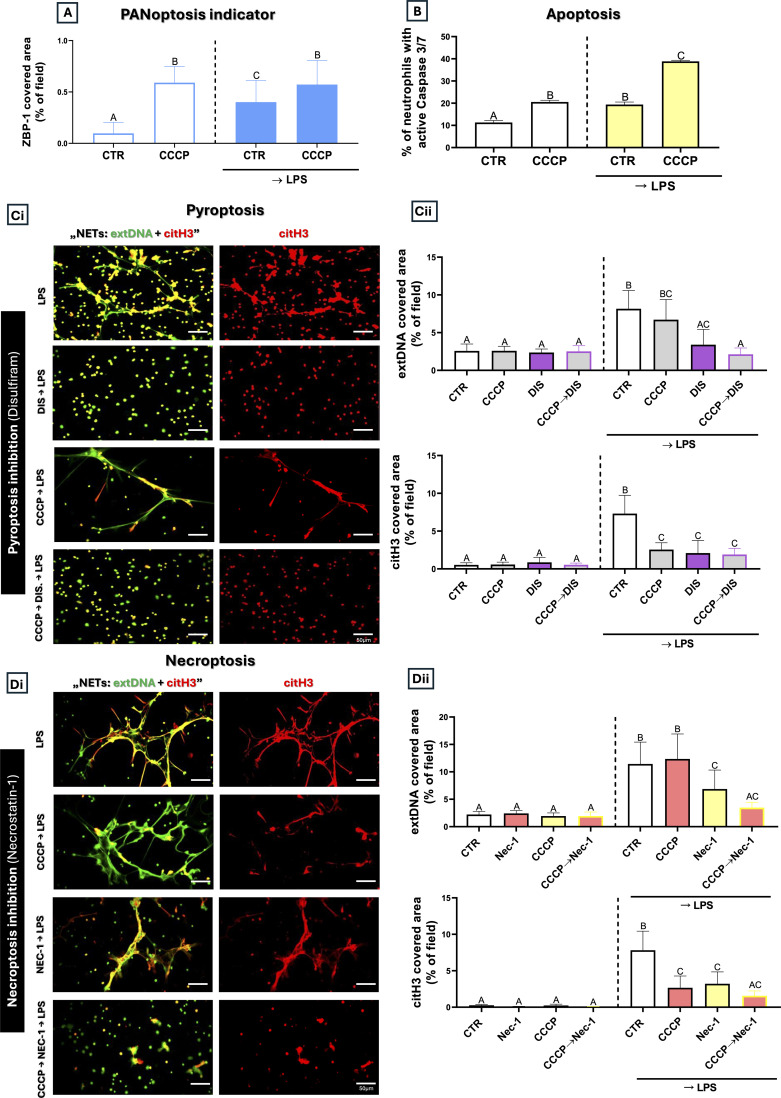
Effect of CCCP possibly leading to lethal NETosis. mitochondria fragmentation inducer on PANoptosis of neutrophils. **(A)** Quantification of ZBP-1 (PANoptosis indicator) expression (confocal microscopy) and **(B)** Caspase 3/7 activity assay (apoptosis; flow cytometry) respectively. Neutrophils were left unstimulated (CTR), stimulated with LPS (50 µg/mL), pre-treated with CCCP (5 µM, 1 h), or pre-treated with CCCP followed by LPS stimulation (CCCP→LPS). To determine pyroptosis **(Ci, Cii)** and necroptosis **(Di, Dii)** extDNA was quantified after CCCP, application of specific inhibitors: Nec-1, DIS treatment (1h) or their combination. Additionally some cells were pretreated with CCCP (CCCP→Nec-1/DIS) incubated with or without LPS. The results are expressed as the mean values ± SD; n≥3. Values significantly different between the groups (p < 0.05) according to one-way ANOVA (*post hoc* Bonferroni test) or Kruskal-Wallis test with Dunn’s *post hoc* are designated by letters, where the same letter indicates no differences between groups (different letters indicate statistical differences. Explanation of abbreviations: ZBP-1, Z-DNA-binding protein 1; extDNA, extracellular DNA; Nec-1, necrostatin-1; DIS, disulfiram.

To better understand the mechanisms underlying extDNA release in CCCP-treated cells, we analyzed in greater detail the roles of individual pathways regulated by distinct types of programmed cell death. We observed that CCCP alone, similar to LPS, increased expression of ZBP1, a key regulator of PANoptosis; however, the effect was much more significant (**Figure 8A**). Subsequently, we confirmed involvement of each of PANasptosis-related cell deaths after CCCP treatment: **(i)** apoptosis - manifested by increased Caspase 3/7 levels (with or without LPS) (**Figure 8B**), **(ii)** pyroptosis **–** by prevention of extDNA when the pathway was inhibited (**Figure 8Ci, Cii****),** and **(iii)** necroptosis - when extDNA release was reduced after this cell death was blocked (**Figure 8Di, Dii**).

## Discussion

NETs are critical effectors of host defense (1). Their formation depends on PAD4-mediated histone citrullination (37), as confirmed by the inability of *Pad4^−/−^* neutrophils to generate NETs in our system. Although PAD4 inhibitors such as Cl-amidine are widely used to block NET formation (44), their limited specificity raises concerns about broader effects on neutrophil functioning. Pathological NET accumulation is well documented in sepsis (8, 45), and their excessive formation and impaired clearance promote endothelial damage, microvascular occlusion, and organ failure. Emerging evidence indicates that NETs can persist in the vasculature and undergo self-renewal, sustaining inflammation long after the initial insult (13). Beyond acute inflammation, NETs are increasingly implicated in cancer and autoimmune diseases, such as multiple sclerosis (MS) (46). Circulating NETs and associated proteins such as neutrophil elastase (NE) and myeloperoxidase (MPO) are elevated in MS patients and correlate with clinical disability and lesion burden, suggesting that chronic inflammation primes neutrophils towards exaggerated NET responses (47, 48). Dimethyl fumarate (DMF), a first-line therapy for relapsing-remitting MS (49), is an ester of the Krebs cycle intermediate fumarate and possesses well-established immunomodulatory properties. As fumarate itself functions as an endogenous immunometabolite, DMF provides a conceptual link between mitochondrial metabolism and immune regulation. Moreover, DMF was shown to exert anti-inflammatory properties in macrophages (23).

Based on these converging lines of evidence, we hypothesized that DMF may function as an immunometabolic inhibitor of NET formation. While initial studies have suggested that DMF can suppress NET release (50, 51), these reports relied predominantly on PMA as a stimulus - a non-physiological activator that bypasses surface receptors and does not accurately recapitulate inflammatory or infectious conditions. Moreover, NET identification in these studies was primarily restricted to extDNA staining (with Sytox), without concurrent assessment of key NET components such as citrullinated histones or neutrophil elastase, the latter being a major mediator of tissue injury during sepsis (8). In the present study, we therefore investigated the effects of DMF on NET formation using a physiologically relevant LPS-driven model and comprehensive NET characterization. We demonstrate that DMF effectively suppresses LPS-induced NET formation, as reflected by strongly reduced extDNA release and diminished citH3 and NE. This effect phenocopies genetic PAD4 deficiency, thereby establishing DMF as a potent inhibitor of PAD4-dependent NET formation under inflammatory conditions. Importantly, statistics with PAD4-deficient mice excluded a possibility that DMF induces NET-independent extDNA or citH3 release.

Multiple studies have demonstrated that Nrf2 activation coordinates cytoprotective programs that link redox control, mitochondrial homeostasis, and inflammation resolution (52). In macrophages, O’Neill and colleagues showed that DMF-driven Nrf2 activation promotes a pro-resolving phenotype and alters inflammatory output, providing a conceptual framework for Nrf2-mediated immune regulation beyond antioxidant gene induction (15, 23). Importantly, recent work has identified mitochondrial dynamics as a key downstream target of Nrf2 signaling. Constitutive Nrf2 activation *via* Keap1 inhibition, as well as pharmacological activation with sulforaphane or DMF, was shown to induce Drp1 degradation and promote a hyperfused mitochondrial network in fibroblast and neuronal cultures (53), establishing a direct link between Nrf2 activity and suppression of pathological mitochondrial fission. Against this backdrop, our findings place neutrophils within this emerging Nrf2-mitochondrial dynamics axis. In our studies, pharmacological inhibition of Nrf2 completely abolished DMF’s ability to suppress NET formation, restoring NET release to levels observed after LPS stimulation alone. Notably, Nrf2 inhibition was effective when applied after DMF treatment, supporting a model in which sustained Nrf2 activity is required to maintain a NET-restrained state. These observations are consistent with our earlier work (20), which demonstrates that the endogenous itaconate derivative 4-OI suppresses NET formation through Nrf2 activation, further supporting the notion that Nrf2 functions as a central checkpoint limiting excessive neutrophil effector responses.

Among pro-resolving mediators linked to Nrf2-dependent immunoregulation, Annexin A1 (ANXA1) has emerged as a key modulator of neutrophil behavior. In neutrophils, ANXA1 is stored in gelatinase granules and rapidly mobilized to the cell surface or extracellular space upon activation (41), where engagement of the formyl peptide receptor 2 (Fpr2) limits neutrophil activation and promotes resolution (54). Functional relevance of this pathway has been demonstrated in human neutrophils, where blockade of FPR2 enhanced NET formation, while treatment with the ANXA1-derived peptide Ac2–26 significantly reduced NET release (55), establishing ANXA1–FPR2 signaling as a negative regulator of NET formation. In macrophages, it was demonstrated that Nrf2 activation by DMF promotes secretion of the 33-kDa cleaved form of ANXA1, which mediates anti-inflammatory effects (23). In our system, DMF increased extracellular ANXA1 levels in LPS-stimulated neutrophils, and pharmacological blockade of Fpr2 (with WRW4) fully reversed the inhibitory effect of DMF on NET formation. Importantly, inhibition of Nrf2 did not significantly reduce ANXA1 release, indicating that ANXA1–Fpr2 signaling is functionally required for NET suppression, regardless of Nrf2. The thirty-three kDa-cleaved form of ANXA1 appears to be a critical step for ANXA1 activity in macrophages (23). In neutrophils, by contrast, secretion of the 33-kDa ANXA1 requires proteolytic processing by elastase (56), metalloprotease (57) or proteinase 3 (58). Importantly, we demonstrated that DMF suppresses neutrophil elastase, making it unlikely that DMF promotes the secretion of the 33-kDa ANXA1 species in neutrophils. Nevertheless, DMF still increases extracellular ANXA1 levels, and *via* Fpr2 engagement, which restrains NET formation, indicating that Nrf2-dependent NET inhibition operates primarily independently of the canonical cleavage-dependent ANXA1 pathway observed in macrophages. Determining which ANXA1 species is functionally responsible for limiting NET formation in this context will require further investigation.

Given that fumarate is a mitochondrial-derived metabolite (22), we also examined whether the inhibitory effect of dimethyl fumarate (DMF) on NET formation involves alterations in mitochondrial integrity and dynamics. In a pediatric porcine model of in-hospital cardiac arrest, DMF administration restored mitochondrial morphology. It mitigated mitochondrial injury following severe ischemic stress, underscoring its capacity to counteract pathological mitochondrial remodeling (59). Similarly, in murine models of Friedreich’s ataxia, DMF dose-dependently increased mitochondrial gene expression and improved mitochondrial function in metabolically active tissues such as muscle and brain (60). Consistent with these observations, we found that inflammatory stimulation induced Drp1 phosphorylation in neutrophils, indicative of enhanced mitochondrial fission, whereas DMF markedly reduced Drp1 activation. Importantly, this effect was accompanied by a more interconnected staining pattern of the mitochondrial fusion markers MFN1/2, and OPA1, further supporting a shift in mitochondrial dynamics in DMF-treated cells. In sepsis, excessive mitochondrial fission has been reported across multiple tissues (61) and is associated with impaired mitochondrial function, increased cell death, and organ failure (29, 30). In our studies, excessive mitochondrial fragmentation, induced by the uncoupler CCCP, was incompatible with NET formation. Conversely, inhibition of mitochondrial fission with Mdivi-1 (a fusion promoter) phenocopied the effect of DMF and restrained NET release, in line with evidence that pharmacological Drp1 inhibition restores mitochondrial architecture and improves cellular viability in experimental sepsis (62), underscoring the pathological relevance of dysregulated mitochondrial dynamics. These findings suggest that mitochondrial fusion-favoring conditions limit NET formation in neutrophils. Notably, our results are consistent with previous reports showing that DMF promotes Drp1 degradation and induces mitochondrial hyperfusion in fibroblasts and neuronal cells (53), extending this concept to neutrophils and identifying mitochondrial dynamics as a conserved target of DMF-mediated immunomodulation. Moreover, pathological mitochondrial fragmentation has also been linked to the release of mitochondrial DNA (mtDNA) (31), a potent pro-inflammatory signal implicated in certain forms of NET formation. Several studies (3, 63) propose that mtDNA can act as a structural or signaling component of NETs during so-called vital NETosis, a process characterized by rapid DNA extrusion from viable neutrophils without plasma membrane rupture (35). Although the term is rarely used today (64), it refers to NET release that is not associated with neutrophil death. Pilsczek and colleagues (43) demonstrated that in vital NETosis, nuclei undergo rounding and decondensation, followed by separation of the inner and outer nuclear membranes, which form bleb-like structures; vesicles containing nuclear DNA are released extracellularly *via* exocytosis to form NETs (47). Our ultrastructural analyses are consistent with this model: following LPS stimulation, neutrophils displayed vesicle-like structures that may contain DNA, supporting the occurrence of vital NETosis in our system. Importantly, pro-inflammatory stimulation induced Drp1 phosphorylation in neutrophils, which became detectable at 1 h and peaked at 3 h after LPS, thereby guiding both mitochondrial mass measurements and TEM analyses at this time point. No Drp1 phosphorylation was detected at very early time points (5–30 min) or after prolonged stimulation (6 h). In contrast to this early mitochondria-associated phase, prolonged stimulation favors lytic NETosis, characterized by NOX-dependent ROS production and plasma membrane rupture (42, 65). Consistently, in our studies, inhibition of NOX activity with DPI suppressed NET formation at later time points, and Live-or-Dye™ staining confirmed loss of membrane integrity coinciding with extDNA at 6 h after LPS stimulation. Notably, CCCP-induced mitochondrial fragmentation did not promote NET release but instead led to features of catastrophic cell death, indicating a diversion away from NETosis toward alternative inflammatory death pathways.

Mitochondrial depolarization induced by CCCP resulted in extDNA release; however, recent studies have linked S100A8/A9^hi neutrophils and excessive mitochondrial fission to ZBP1 activation and PANoptosis (39) - a coordinated inflammatory cell death program integrating pyroptosis, apoptosis, and necroptosis (38) which has been implicated in neutrophil-driven endothelial injury (39). Consistent with this framework, CCCP-induced mitochondrial collapse in neutrophils was accompanied by caspase activation and engagement of pyroptotic and necroptotic signaling pathways. Inhibition of these pathways significantly reduced extDNA release, indicating that DNA extrusion under severe mitochondrial stress reflects inflammatory cell death rather than regulated NET formation. Notably, extDNA release following CCCP treatment occurred independently of PAD4, supporting a mechanism distinct from canonical chromatin decondensation. Emerging work demonstrates that in neutrophils, pyroptotic and necroptotic pathways potentially involve gasdermin family pore formation (66, 67) can promote membrane permeabilization and likely extracellular DNA (extDNA) release without the formation of classical NET structures. Our ultrastructural analyses further supported this interpretation, revealing mitochondrial damage, mitophagy, and vacuolization after CCCP treatment. In contrast, DMF suppressed mitochondrial stress-induced ZBP1 activation and downstream inflammatory cell death signaling without inducing neutrophil death, indicating that DMF restrains NET formation by actively regulating mitochondrial integrity rather than diverting cells toward PANoptotic death.

Collectively, our data support a model in which DMF restrains pathological NET formation through coordinated regulation of mitochondrial dynamics, Nrf2-dependent signaling, and engagement of ANXA1–Fpr2 pathways. These findings identify mitochondrial integrity as a critical checkpoint in neutrophil fate decisions and position immunometabolic modulation as a promising strategy to limit NET-driven pathology without broadly suppressing innate immune function.

## Data Availability

The original contributions presented in the study are included in the article/**Supplementary Material**. Further inquiries can be directed to the corresponding author.
