# Supplementary material for: Fumarate inhibits the formation of neutrophil extracellular traps (NETs) in a Nrf2-controlled and Annexin-A1-dependent manner associated with mitochondrial fusion
Source: Front Immunol. 2026 Mar 31;17:1770063. doi: 10.3389/fimmu.2026.1770063 (PMC13076181; doi:10.3389/fimmu.2026.1770063)
Supplement: Supplementary file 1 [file DataSheet1.pdf]

Supplementary Figure 1

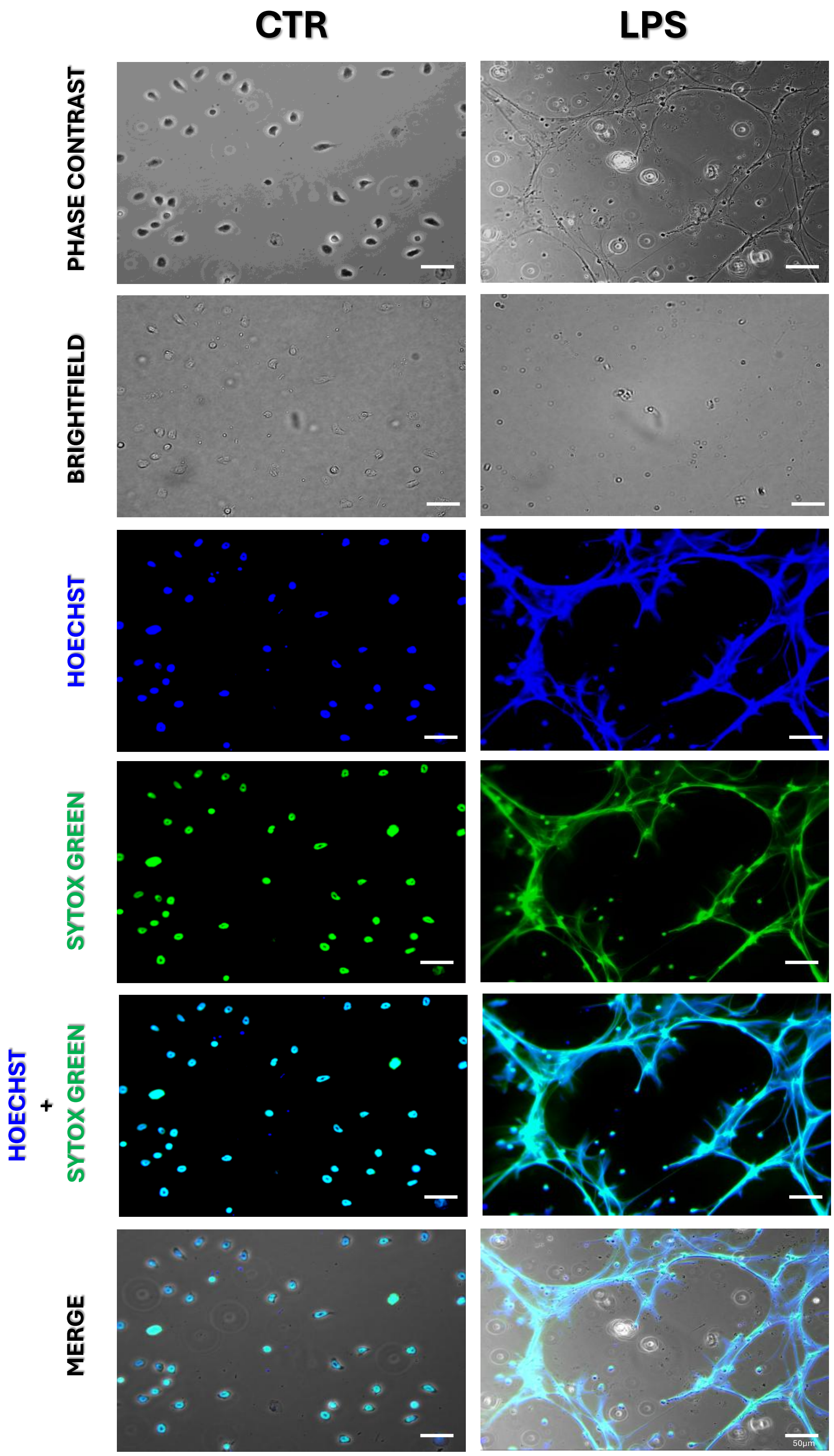

Supplementary Figure 1 Validation of Sytox Green as a nuclear DNA marker in fixed neutrophils. Neutrophils were fixed and either incubated with lipopolysaccharide (LPS) at a concentration of 50 µg/ml (for 6 h) or left unstimulated (CTR) then stained in parallel with Hoechst (blue) and Sytox Green (green). Representative images acquired in phase contrast and brightfield are shown alongside fluorescence channels. To visualize co-localization of extDNA/cell nucleus stained with either Hoechst or Sytox Green, the images from each channel were overlaid.

Supplementary Figure 2

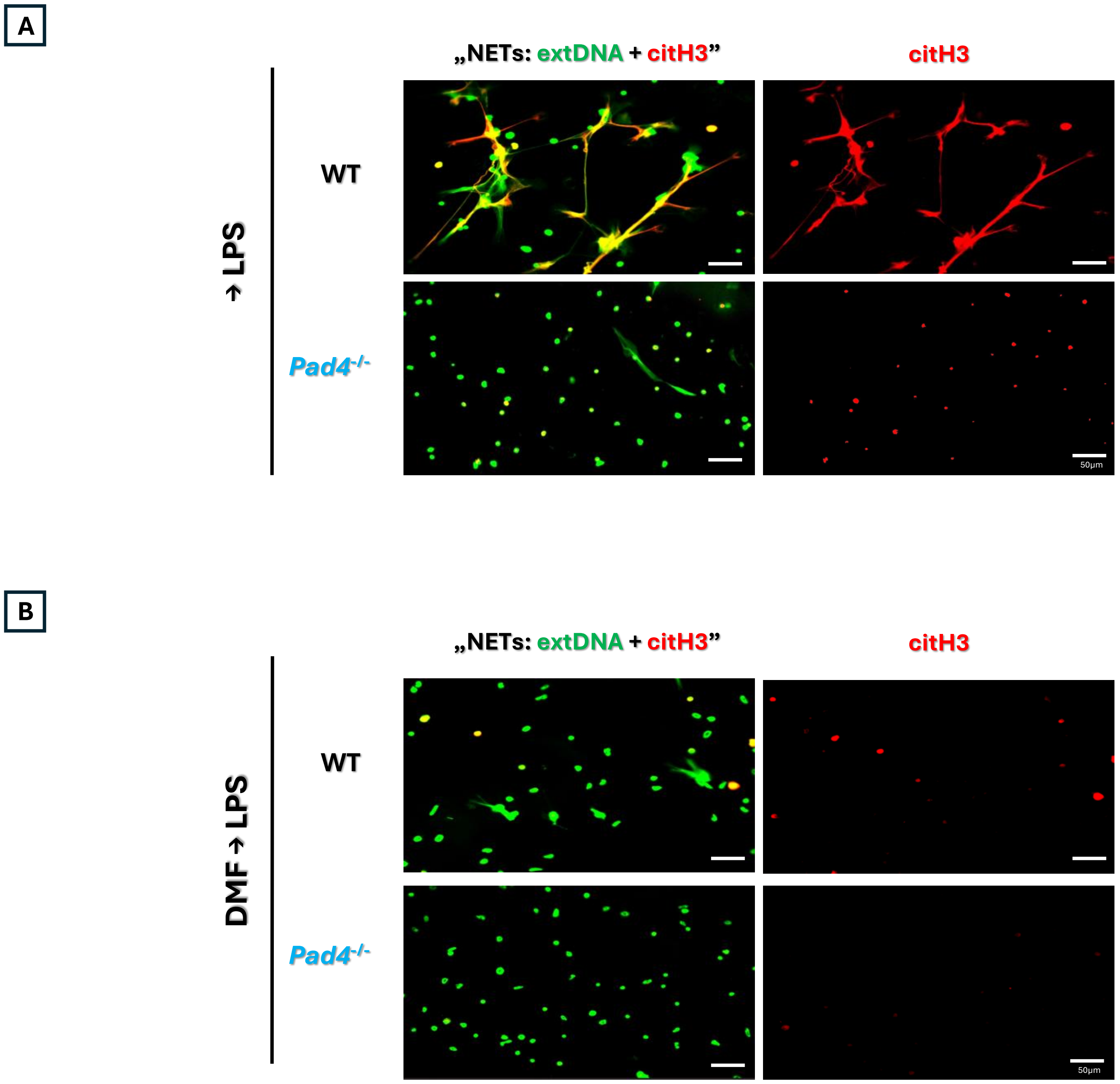

Supplementary Figure 2 Comparison of NETs released by neutrophils isolated from wild-type (WT) and peptidylarginine deiminase 4 KO (*Pad*4$^{-/-}$) mice in response to lipopolysaccharide (LPS) and dimethyl fumarate (DMF). Neutrophils were either incubated with lipopolysaccharide (LPS) at a concentration of 50 µg/mL (for 6 h) or pre-treated with DMF (25 µM; 1 h) and stimulated with LPS (DMF→LPS). Representative images of NETs formed by LPS alone **(Ai)** or in the presence DMF **(B)** are shown in extDNA in green while citH3 is in red. To visualize co-localization of NET components, the images from each channel were overlaid (NETs: extDNA + citH3). Explanation of abbreviations: extDNA - extracellular DNA, citH3 - citrullinated histone H3

Supplementary Figure 3

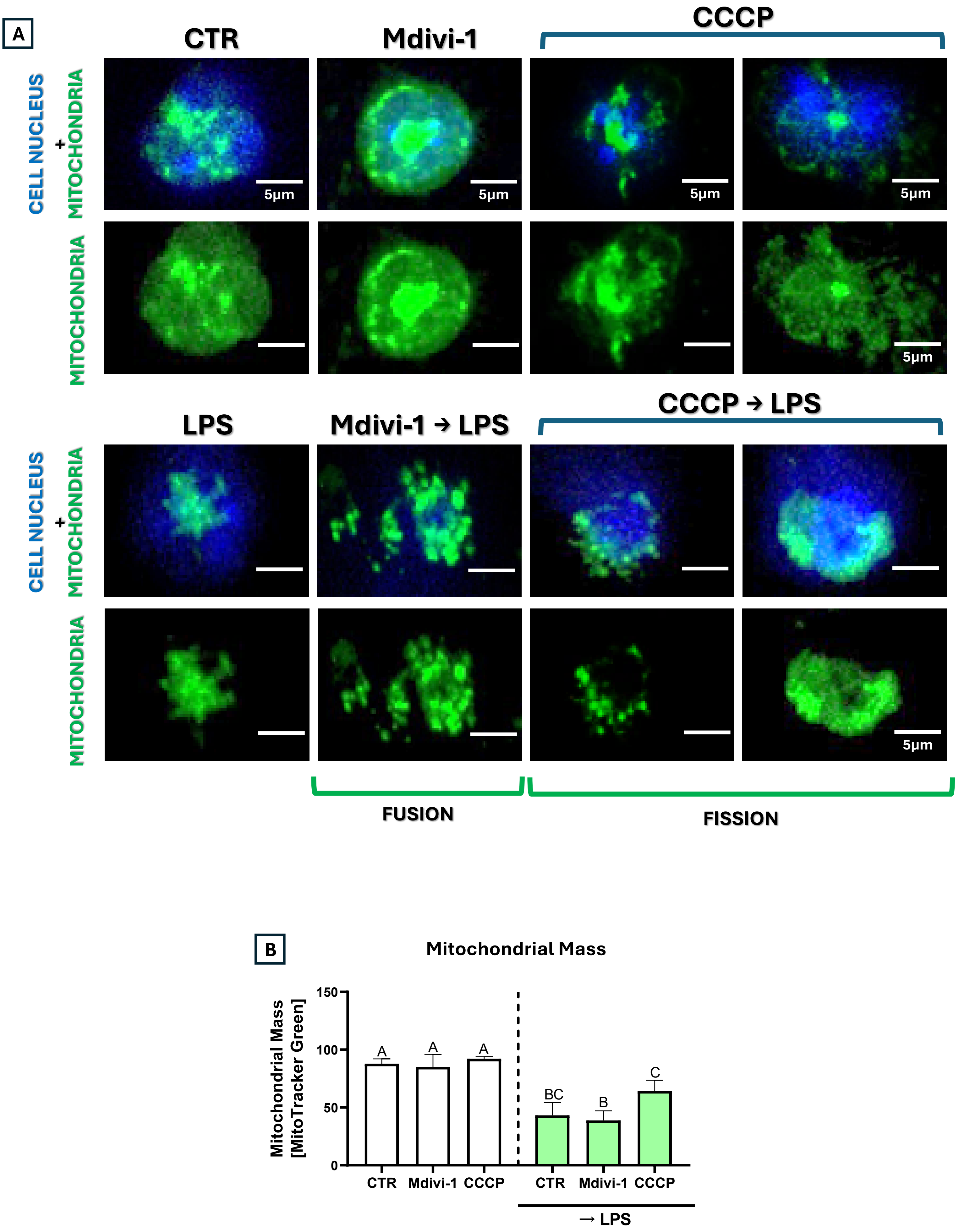

Supplementary Figure 3 Visualization of mitochondria dynamics induced by mdivi-1 (fusion) and CCCP (fission/fragmentation). **(A)** Representative images of neutrophils pre-treated with mdivi-1 (20µM) or CCCP (5 µM) for 1 h, incubated with lipopolysaccharide (LPS) at a concentration of 50 µg/mL (for 3 h), pre-treated with mdivi-1/CCCP and stimulated with LPS (mdivi-1/CCCP→LPS) or left unstimulated (CTR). Mitochondria (MitoTracker) are shown in green while cell nucleus (Hoechst) in blue. **(B)** Measurement of Mitochondrial Mass with MitoTracker Green in flow cytometry upon the above treatments. The results are expressed as the mean values ± SD; n≥3. Values significantly different between the groups ($p < 0.05$) according to one-way ANOVA (*post hoc* Bonferroni test) are designated by letters, where the same letter indicates no differences between groups (different letters indicate statistical differences.

Supplementary Figure 4

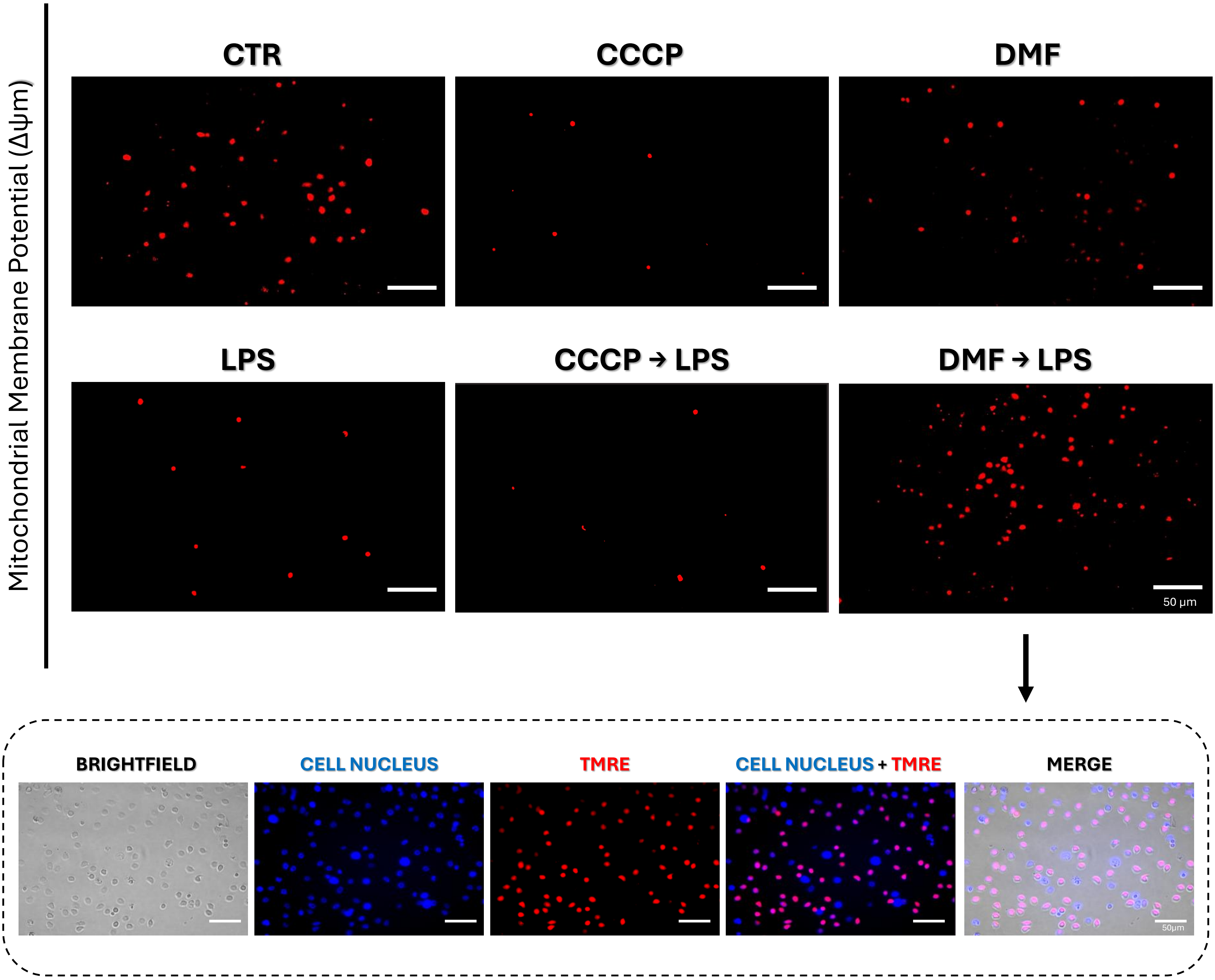

Supplementary Figure 4 State of mitochondrial membrane potential (ΔΨm) after mitochondria dynamics changes assessed by the tetramethylrhodamine ethyl ester perchlorate (TMRE). Representative images of neutrophils pre-treated with DMF (25 μM) or CCCP (5 μM) for 1 h, incubated with lipopolysaccharide (LPS) at a concentration of 50 μg/mL (for 3 h), pre-treated with DMF/CCCP and stimulated with LPS (DMF/CCCP→LPS) or left unstimulated (CTR). Mitochondria ΔΨm assessed by incubation with TMRE for 30 min is shown in red. For the DMF→LPS group, representative brightfield, cell nucleus (Hoechst), TMRE, cell nucleus (Hoechst) + TMRE (merged), and full merged images are additionally provided to facilitate visualization of cellular morphology and signal localization.

Supplementary Figure 5

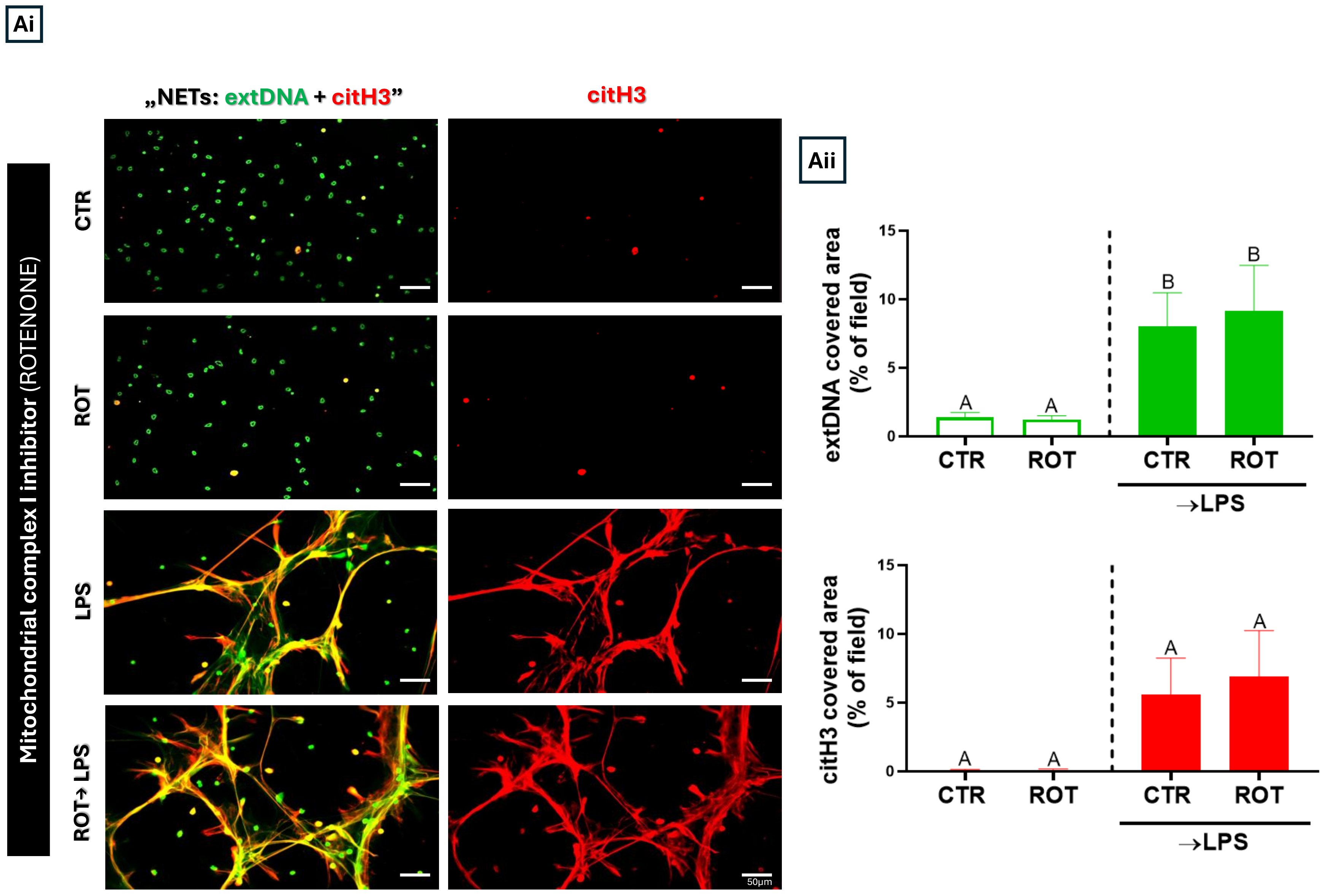

Supplementary Figure 5 Effect of mitochondrial complex I inhibition by rotenone on neutrophil extracellular trap (NET) formation. Neutrophils were pre-treated with rotenone (ROT; 5 µM) for 1 h or incubated with lipopolysaccharide (LPS) at a concentration of 50 µg/mL (for 6 h), pre-treated with ROT and stimulated with LPS (ROT→LPS) or left unstimulated (CTR). Representative images of NETs formed by LPS in the presence of ROT are shown in **(Ai)**: extDNA - green, citH3 - red. To visualize co-localization of NET components, the images from each channel were overlaid (NETs: extDNA + citH3). **(Aii)** Quantification of NET formation: area covered by the extDNA and citH3 signal. The results are expressed as the mean values ± SD; $n \geq 3$. Values significantly different between the groups ($p < 0.05$) according to one-way ANOVA (*post hoc* Bonferroni test) are designated by letters, where the same letter indicates no differences between groups (different letters indicate statistical differences. Explanation of abbreviations: extDNA - extracellular DNA, citH3 - citrullinated histone H3

Supplementary Figure 6

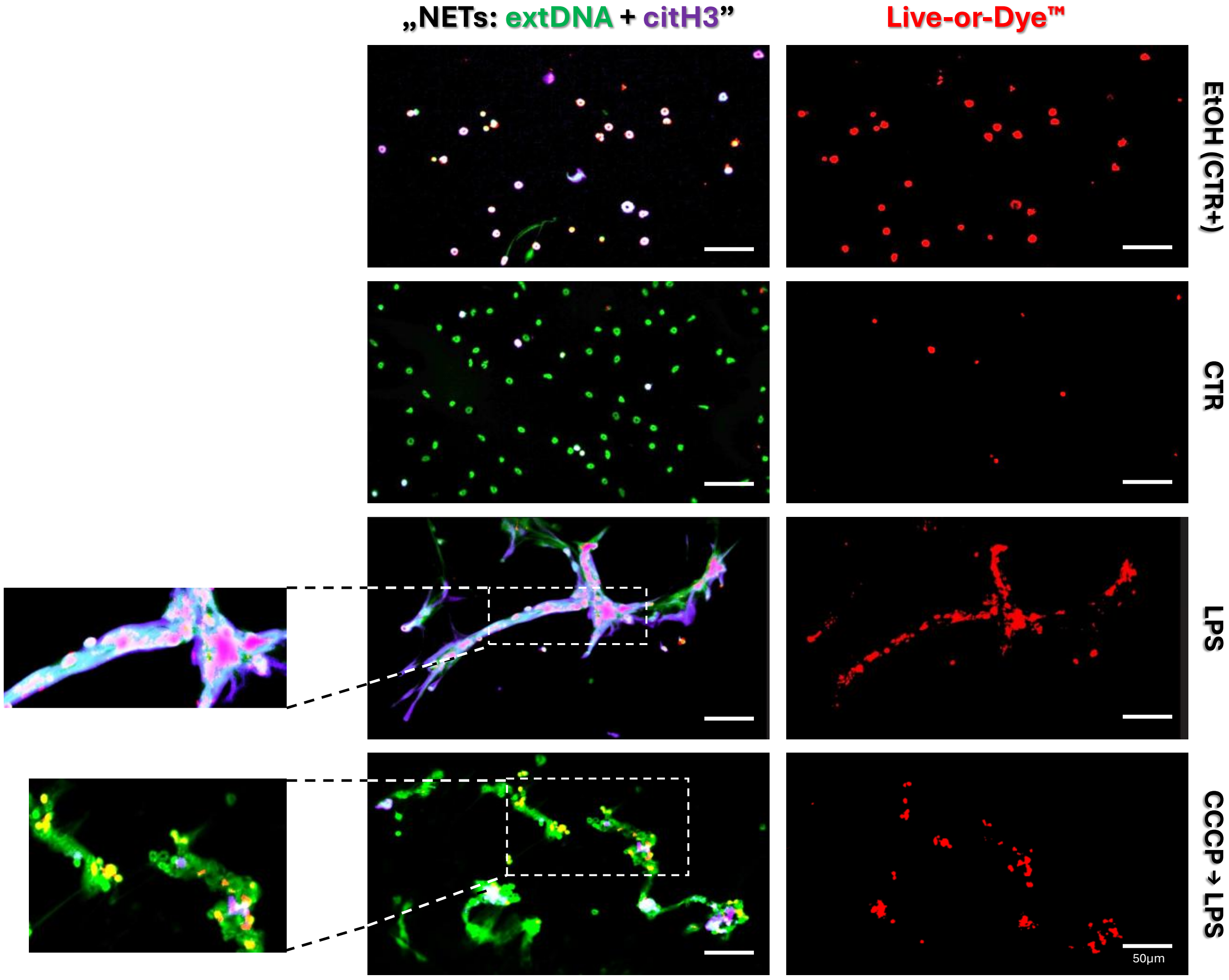

Supplementary Figure 6 Effect of lipopolysaccharide (LPS) and mitochondria fragmentation (CCCP) on the NETosis type (lytic/vital). Neutrophils were either pre-treated with CCCP (5 µM; for 1 h), incubated with lipopolysaccharide (LPS) at a concentration of 50 µg/mL (for 6 h), pre-treated with CCCP and stimulated with LPS (CCCP→LPS) or left unstimulated (CTR). As a positive control (CTR+) indicating cell death - EtOH was used. Representative images of NETs formed by LPS in the presence or absence of CCCP are shown as extDNA in green, citH3 in violet and prefixable Live-or-Dye™ used for lytic NETosis labeling in red. To visualize co-localization of NET components, the images from each channel were overlaid (NETs: extDNA + citH3). Explanation of abbreviations: extDNA - extracellular DNA, citH3 - citrullinated histone H3

Supplementary Figure 7

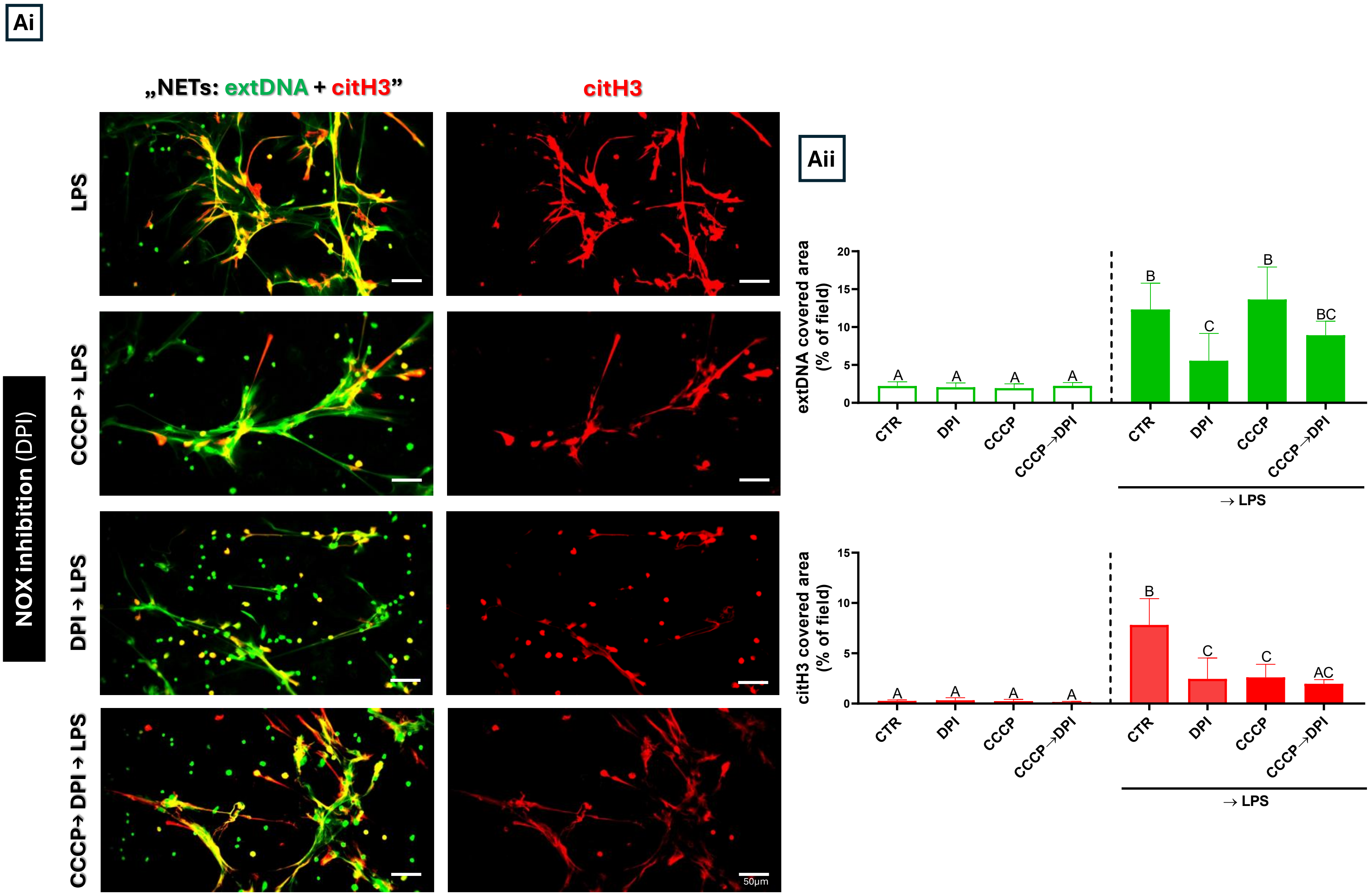

Supplementary Figure 7 Effect of mitochondria fission/fragmentation induced by CCCP on the NOX-dependent formation of neutrophil extracellular traps (NETs). Neutrophils were either pre-treated with diphenyleneiodonium chloride (DPI; 10 µM) or CCCP (5 µM) for 1 h or their combination: CCCP with DPI - CCCP→DPI→LPS incubated with lipopolysaccharide (LPS) at a concentration of 50 µg/mL (for 6 h), pre-treated with DPI/CCCP and stimulated with LPS (DPI/CCCP→LPS) or left unstimulated (CTR). Representative images of NETs formed by LPS in the presence of DPI/CCCP are shown in **(Ai)**: extDNA - green, citH3 - red. To visualize co-localization of NET components, the images from each channel were overlaid (NETs: extDNA + citH3). **(Aii)** Quantification of NET formation: area covered by the extDNA and citH3 signal. The results are expressed as the mean values ± SD; n≥3. Values significantly different between the groups ($p < 0.05$) according to one-way ANOVA (*post hoc* Bonferroni test) or Kruskal-Wallis test with Dunn's *post hoc* are designated by letters, where the same letter indicates no differences between groups (different letters indicate statistical differences. Explanation of abbreviations: extDNA - extracellular DNA, citH3 - citrullinated histone H3
